# Open-label randomised controlled trial of aripiprazole/sertraline combination in comparison with quetiapine for the clinical and cost-effectiveness of treatment of bipolar depression (the ASCEnD study): study protocol

**DOI:** 10.1136/bmjopen-2025-112677

**Published:** 2026-03-19

**Authors:** Lumbini Azim, Sarah Al-Ashmori, Chrissie Butcher, Andrea Cipriani, Carolyn A Chew-Graham, Emily Clare, Emma Clark, Michael Cole, Susanna Carella, Lyndsey Dixon, Jonathan Evans, Tania Gergel, John Gibson, Helen C Hancock, Isobel Hoppe, David Kessler, Thomas Kabir, Glyn Lewis, Ayesha Mathias, Richard Morris, Neil Nixon, Judit Simon, Marion Dawn Teare, The Lived Experience Advisory Panel, Fiona Cammack, Mourad Wahba, Lauren Wall, Zoe Walmsley, Dennis Wienand, Faye Wolstenhulme, Niraj Ahuja, Stuart Watson

**Affiliations:** 1 Cumbria Northumberland Tyne and Wear NHS Foundation Trust, Newcastle upon Tyne, UK; 2 Northern Centre for Mood Disorders, Newcastle University Translational and Clinical Research Institute, Newcastle upon Tyne, UK; 3 Biostatistics Research Group, Newcastle University Population Health Sciences Institute, Newcastle upon Tyne, UK; 4 Newcastle Clinical Trials Unit, Newcastle upon Tyne, UK; 5 Oxford Health NHS Foundation Trust, Oxford, UK; 6 University of Oxford Department of Psychiatry, Oxford, UK; 7 Keele University School of Medicine, Keele, UK; 8 Avon and Wiltshire Mental Health Partnership NHS Trust, Bath, UK; 9 Bristol Medical School, Bristol, UK; 10 Bipolar UK, University College London Division of Psychiatry, London, UK; 11 Cardiff University Division of Psychological Medicine and Clinical Neurosciences, Cardiff, UK; 12 The McPin Foundation, London, UK; 13 University College London Division of Psychiatry, London, UK; 14 Mental Health and Clinical Neurosciences, University of Nottingham School of Medicine, Nottingham, UK; 15 Nottinghamshire Healthcare NHS Foundation Trust, Nottingham, UK; 16 Medical University of Vienna Department of Health Economics, Vienna, Austria

**Keywords:** Bipolar and Related Disorders, Depression & mood disorders, Adult psychiatry, Clinical Protocols, Randomized Controlled Trial, HEALTH ECONOMICS

## Abstract

**Introduction:**

Bipolar disorder affects around 2% of the population and is linked with reduced life expectancy and socioeconomic burden. Depressive episodes are difficult to treat and typically more prevalent, enduring and burdensome than manic episodes. The use of antidepressants alone has limited effect and is associated with significant clinical risk through polarity switch. Current National Institute for Health and Care Excellence guidelines recommend quetiapine, olanzapine (with or without fluoxetine) and lamotrigine; however, these medications have limited efficacy, tolerability and acceptability. The ASCEnD study aims to assess the clinical and cost-effectiveness of aripiprazole plus sertraline compared with quetiapine, offering potential improvements for outcomes in bipolar depression. The study is funded by the National Institute for Health and Care Research Health Technology Assessment programme (NIHR132773).

**Methods and analysis:**

ASCEnD is a prospective, two-arm, superiority, individually 1:1 randomised, controlled, pragmatic, parallel group, type A open-label clinical trial of aripiprazole/sertraline medication combination compared with quetiapine for bipolar depression. The study is conducted in the UK National Health Service setting with the aim of recruiting and randomising 270 participants followed-up for 24 weeks. Adults with bipolar disorder self-refer or are recruited through primary and secondary care services. The primary outcome is change in depressive symptoms 12–16 weeks after randomisation. Secondary outcomes include measures of symptom change, treatment satisfaction, tolerability, medication adherence, concomitant medication use, psychosocial functioning, quality of life and cost-effectiveness and informal carer measures of quality of life and costs of caring. The exploratory outcome is change in participant reward and punishment responsiveness. Analysis will follow a prespecified statistical analysis plan. A nested qualitative study is included to examine feasibility and acceptability of the trial design.

**Ethics and dissemination:**

A Clinical Trial Authorisation from Medicines and Healthcare products Regulatory Agency, and approval from the Health Research Authority (IRAS 1007468) and North East – Newcastle and North Tyneside 1 Research Ethics Committee (23/NE/0132) were obtained. Results will be disseminated through peer-reviewed publications, conference presentations and lay summaries for participants and patient and public groups.

**Trial registration number:**

ISRCTN63917405.

**STRENGTHS AND LIMITATIONS OF THIS STUDY**

- The trial addresses a key evidence gap for treatment of bipolar disorder with antipsychotic/antidepressant combinations.
- The pragmatic comparison of accessible and cost-effective medications is clinically relevant for translation of findings into practice.
- The open-label design precluded blinding and may influence participant-reported outcomes across treatment arms.

## Introduction

### Background and rationale

 Bipolar disorder, characterised by episodes of mania or hypomania, depression and mixed states, is a chronic and debilitating condition with an annual prevalence estimated at 0.8% in the UK.^1^ The impact of bipolar disorder on mortality is also greater than commonly considered risk factors such as smoking^2^ with life expectancy reduced by 13 years^3^ and an increasing mortality gap of 10–20 years, particularly in younger populations with bipolar disorder.^4^ The economic cost is also significant, estimated at £6.43 billion annually in the UK in 2018, including lost productivity, informal care and healthcare expenses.^1^

People with bipolar disorder are symptomatic around half of the time, the vast majority of which is depression.^5^,^8^ Depressive episodes in bipolar disorder—referred to as ‘bipolar depression’—are often persistent, with 50% of people remaining depressed at 6 months.^9^ Compared with manic episodes, depressive episodes exert a greater burden on caregivers, quality of life, education, occupation, economic costs and suicide risk.^8 10 11^ Depressive episodes are also associated with higher hospitalisation costs which may reflect greater symptom persistence and treatment complexity compared with manic episodes.^12^

Bipolar depression does not respond as effectively to conventional psychological interventions recommended for major depressive disorder (MDD).^13^,^15^ Current National Institute for Health and Care Excellence (NICE) guidelines recommend quetiapine, olanzapine (with or without fluoxetine) and lamotrigine for the treatment of bipolar depression.^13^,^15^ These options have significant limitations, including low tolerability due to side effects such as weight gain and sedation,^16 17^ extension of the corrected QT interval^18 19^ and orthostatic hypotension,^20^ all contributing to medication non-adherence.

There is also limited evidence to support the efficacy of antidepressants in bipolar depression, yet in the UK, approximately 49% of individuals with bipolar disorder are prescribed at least one antidepressant^21^ with selective serotonin reuptake inhibitors being the most common class.^22^ While small studies suggest some evidence of treatment response,^23^,^25^ larger trials^26^ and meta-analyses^27^ indicate that antidepressants alone or in combination with mood stabilisers offer minimal efficacy and pose risks such as mood destabilisation,^28^ chronic dysphoria^29^ and manic switching.^30^,^38^ The only evidenced^16 39^ and recommended^13^,^1540^ antipsychotic/antidepressant combination with proven efficacy is olanzapine/fluoxetine, yet it is rarely prescribed in practice due to tolerability issues.^3947^,^50^ This evidence-practice gap also illustrates the clinical uncertainty about the use of antidepressants in bipolar depression^51 52^ and has been highlighted as a critical research gap by both NICE and the James Lind Alliance.^53^ Aside from olanzapine/fluoxetine in combination, other antipsychotic/antidepressant combinations have not been tested in adequately powered trials in bipolar depression. It is therefore unknown whether there is something unique about the specific combination of olanzapine with fluoxetine or whether alternative antipsychotics and antidepressants might also be effective when combined.

Aripiprazole has unique pharmacological properties that may enhance antidepressant efficacy^54^,^61^ and prevent the chance of manic switch^62 63^; it modulates dopamine activity,^64^ thereby facilitating reward activation,^65 66^ enhances serotonin neurotransmission^67^,^70^ and has established efficacy as an adjunct treatment in MDD.^54^,^61^ Aripiprazole augmentation in unipolar depression uses lower doses (≤5 mg/day) compared with the higher doses used to treat psychotic symptoms (15–30 mg/day) and is associated with significant symptom reduction.^59^,^6171 72^ Preliminary evidence also suggests potential benefits in bipolar depression,^73^,^82^ particularly at lower doses where akathisia risk is also minimised.^83^

Sertraline has been chosen as the antidepressant to combine with aripiprazole due to its strong safety profile, a favourable pharmacokinetic profile, limited interactions with most psychotropics and lack of adverse interaction with aripiprazole.^84^ It is well-tolerated in both bipolar^23^ and unipolar^85^ depression populations and does not carry a risk of cardiac arrhythmias.^86^,^89^ Augmentation with low doses of aripiprazole may also amplify response to sertraline via dopaminergic and serotonergic modulation.^67^

Quetiapine is the preferred comparator due to its robust evidence base for treating bipolar depression, indicated by symptom reduction and improvement in overall functioning.^2690^,^92^ It is one of the few NICE-recommended treatments for bipolar depression^93^ and widely prescribed in primary care.^50^ Quetiapine monotherapy is also more cost-effective than other treatments for bipolar depression with comparable quality-adjusted life years and lower overall health and social care expenditures.^94^

The ASCEnD trial aims to determine whether an aripiprazole/sertraline combination is more effective and cost-effective in bipolar depression compared with quetiapine monotherapy. Both aripiprazole/sertraline combination and quetiapine monotherapy may be proven as efficacious in the treatment of bipolar depression; however, if the aripiprazole/sertraline combination is better tolerated, it may improve adherence, resulting in utilisation of an effective treatment. This would make the aripiprazole/sertraline combination more clinically effective and potentially more cost-effective than quetiapine,^95 96^ offering a superior alternative that can improve service user outcomes and reduce reliance on secondary care.^97 98^ Given the current delays in accessing specialist mental health services,^99^,^101^ a treatment regimen that can be initiated in primary care would enhance service user accessibility and management across the integrated care system.^102^ Conversely, if quetiapine remains superior, this will reinforce current recommendations against antidepressant use for bipolar depression,^30^,^38^ challenging their prevalent off-label prescription.^50^ Addressing these uncertainties could bridge the evidence-practice gap, inform clinical guidelines and routine practice and improve outcomes for individuals with bipolar depression by identifying a more effective and accessible treatment strategy.

### Objectives

#### Primary objective

- To determine whether the improvement in depression is greater in participants randomised to aripiprazole/sertraline combination than those randomised to quetiapine.

#### Secondary objectives

To compare the impact of aripiprazole/sertraline combination vs quetiapine on:

- The trajectories of symptom change, treatment satisfaction (taken as a measure of ‘treatment acceptability’), tolerability, pattern of medication adherence and pattern of non-randomised antipsychotic/antidepressant medication use.
- Change in anxiety symptoms and manic symptoms, including rates of relapse to a hypomanic or manic episode.
- Psychosocial functioning, health-related quality of life and capability well-being.
- Costs and incremental cost-effectiveness.
- Informal carers’ health-related quality of life.

#### Exploratory objective

- To explore changes in reward and punishment responsiveness.

#### Qualitative objective

- To inform the refinement of study processes by exploring service user and clinician perspectives on trial acceptability.

## Methods and analysis

### Study design

The ASCEnD trial is a prospective, two-arm, superiority, individually 1:1 randomised, controlled, pragmatic, parallel group, type A open-label clinical trial of aripiprazole/sertraline medication combination compared with quetiapine for bipolar depression. Participants are followed-up for 24 weeks after randomisation. The schedule of study procedures is shown in table 1.

**Table 1:** Schedule of events

Procedures	Screening	Baseline	Follow-up week																							
			1	2	3	4	5	6	7	8	9	10	11	12	13	14	15	16	17	18	19	20	21	22	23	24
**Site activities**																										
Informed consent	✓																									
Eligibility assessment	✓*†																									
Pregnancy testing	✓																									
Demographics	✓																									
Medical history	✓																									
Review of current medications	✓																									
Randomisation		✓																								
Prescribe trial medication		✓																								
Participant recompense		✓														✓										✓
**Participant activities**																										
QIDS-SR	✓†		✓	✓	✓	✓	✓	✓	✓	✓	✓	✓	✓	✓	✓	✓	✓	✓	✓	✓	✓	✓	✓	✓	✓	✓
GAD-7, ASRM		✓	✓	✓	✓	✓	✓	✓	✓	✓	✓	✓	✓	✓	✓	✓	✓	✓	✓	✓	✓	✓	✓	✓	✓	✓
Review of current medications			✓	✓	✓	✓	✓	✓	✓	✓	✓	✓	✓	✓	✓	✓	✓	✓	✓	✓	✓	✓	✓	✓	✓	✓
TSQM, GASS, MARS, WSAS, EQ-5D-5L, ICECAP-A, HEQ, OxCAP-MH		✓				✓										✓										✓
PILT, BIS/BAS		✓‡		✓§										✓§												
cRA follow-up			✓	✓	✓	✓	✓	✓	✓	✓	✓	✓	✓	✓	✓	✓	✓	✓	✓	✓	✓	✓	✓	✓	✓	✓
**Informal carer activities**																										
EQ-5D-5L, ICECAP-A, OxCAP-MH, CIIQ		✓¶				✓										✓										✓

* Includes completion of the SCID-5-RV.

† To be completed within 7 days preceding randomisation.

‡ Optional baseline exploratory measures to be completed prior to trial medication initiation.

§ Optional follow-up exploratory measures post-trial medication initiation date. First follow-up to be completed between weeks 3–4 and second follow-up between weeks 13–14.

¶ To be completed within 14 days of the participant’s baseline measures.

ASRM, Altman Self-Rating Mania Scal; BIS/BAS, Behavioural Inhibition System/Behavioural Approach System; CIIQ, Caregiver Indirect and Informal Care Cost Assessment Questionnaire; cRAs, central Research Assistants; GAD-7, General Anxiety Disorder-7; GASS, Glasgow Antipsychotic Side-effect Scale; HEQ, Health Economics Questionnaire; ICECAP-A, ICEpop CAPability measure for Adults; MARS, Medication Adherence Rating Scale; OXCAP-MH, Oxford CAPabilities Questionnaire-Mental Health; PILT, Probabilistic Instrumental Learning Task; QIDS-SR, Quick Inventory of Depressive Symptomatology—Self Report; SCID-5-RV, Structured Clinical Interview for Diagnostic and Statistical Manual-5-Text Revision Disorders-Research Version; TSQM, Treatment Satisfaction Questionnaire for Medication; WSAS, Work and Social Adjustment Scale.

The trial was registered on 23 November 2023 (ISRCTN63917405; https://doi.org/10.1186/ISRCTN63917405). Study protocol V.3.0 (dated 11 September 2024) is reported in accordance with the Standard Protocol Items: Recommendations for Interventional Trials 2025 statement^103^ and the checklist is provided as online supplemental material 1.

### Study setting

The trial is carried out in UK National Health Service (NHS) primary, secondary and tertiary care mental health services. A list of study sites can be obtained from the trial website.^104^

### Eligibility criteria

Eligibility criteria for recruiting participants to the trial are pragmatic, in line with UK clinical practice. There is no upper or lower limit of bipolar depression treatment resistance, duration of current depressive episode, time since initial symptoms or time since diagnosis of bipolar disorder. Service users are not excluded if they are receiving, planning to receive or have recently received psychological or digital therapies.

Inclusion criteria are:

- Aged 18 or over at the point of consent.
- Able to provide written informed consent.
- A current (ie, within 7 days) Diagnostic and Statistical Manual-5-Text Revision (DSM-5-TR)^105^ confirmed diagnosis of a major depressive episode within bipolar disorder. This is confirmed using the Structured Clinical Interview for DSM-5-TR Disorders-Research Version.^106^
- A current (ie, within 7 days) Quick Inventory of Depressive Symptomatology—Self Report (QIDS-SR)^107^ score greater than 10.
- Clinical uncertainty regarding the next course of treatment and judgement that the aripiprazole/sertraline combination and quetiapine treatment arms are both clinically appropriate and represent equipoise.
- In the opinion of the clinician, the participant can follow trial prescription instructions, complete weekly questionnaires and engage in weekly telephone calls with the central Research Assistants (cRAs) throughout the 24-week follow-up period of the trial.

Exclusion criteria are:

- Currently participating in any other interventional clinical trial that may affect the outcome of ASCEnD.
- DSM-5-TR defined severe substance use disorder.
- Any known contraindications to aripiprazole, sertraline or quetiapine.
- Currently pregnant, planning to become pregnant during the trial and/or breastfeeding.

#### Informal carers

Informal carers must be nominated by participants, provide unpaid support to the participant, over the age of 18 and able to complete questionnaires online.

### Sample size

A minimum clinically important change in QIDS-SR of two points was used, as previously established in two relevant clinical trials—PreDiCT^108^ and CEQUEL.^92^ Design parameters calculated from these trials were applied, specifically an SD of 5.4 for the QIDS-SR outcome and a correlation of 14-week QIDS-SR with a baseline of 0.5. Based on PreDiCT and CEQUEL, a conservative estimate of 0.9 correlation between two post-randomisation measures observed between 12 and 16 weeks was assumed to power the trial. The sample size calculation assumed an average of two (of the total five) measurements would be available. An attrition rate (defined as dropping out of the trial follow-up between randomisation and week 12) of 20% was further conservatively assumed for the primary outcome point, based in part on CEQUEL.^92^

Randomising 270 participants would provide 90% power using 5% two-sided significance testing. Including two or more weekly measurements for the repeated measures design increased power; this design enhances the trial efficiency and cost-effectiveness while maintaining the robustness of the evidence of effect size and reducing the required sample size.

### Recruitment

Recruitment efforts prioritise inclusivity, specifically for underrepresented socio-demographic groups. This is supported by Regional Research Delivery Networks (RRDNs) through multiple pathways, including electronic health record searches in primary and secondary care, clinician referrals and opportunistic approaches. Primary care services may send an invitation text message (online supplemental appendix 1) or letter (online supplemental appendix 2) to people identified with bipolar disorder. The study also leverages NIHR Be Part of Research (BPoR)^109^ and Research+Me^110^ national research registries to facilitate participant identification and outreach. BPoR sends an email invitation to volunteers in their registry (online supplemental appendix 3). A targeted digital media campaign organised by the advertising company Just-R facilitates recruitment including self-referral through posters; self-referral is also supported by the charities Bipolar UK and the McPin Foundation, RRDNs, affiliated universities and study sites and the trial website.^104^

Prospective participants may complete an online pre-screening assessment supported by the Research+Me platform. Potentially eligible participants are notified via email and directed to the ASCEnD website containing an online copy of the study participant information sheet (online supplemental appendix 4). Consent can be given to share pre-screening results and contact details with their local study team to facilitate screening appointment arrangements. Interested individuals who do not complete the pre-screening assessment may also be provided with the online or paper (online supplemental appendix 5) participant information sheet.

Informal carers are approached through participant nominations and provided an informal carer information sheet (online supplemental appendix 6).

### Randomisation

Randomisation takes place as soon as possible and no more than 1 week after a participant has been confirmed as eligible. Participants are randomised in a 1:1 ratio to aripiprazole/sertraline combination or quetiapine after their baseline measures are completed. Randomisation incorporates block stratification using three variables: being in mental health secondary care services at screening (Yes/No), being prescribed antidepressants at screening (Yes/No) and being prescribed an antipsychotic at screening (Yes/No). Randomisation is conducted at sites by research staff using Red Pill, which is a central, secure, 24-hour web-based randomisation system with concealed allocation, owned by Sealed Envelope.

### Intervention

Trial medication is provided open-label by local pharmacies according to their usual prescribing practices and any brand of study medication may be used. The study does not mandate use of specific medication dosages or dose escalation procedures. Instead, clinicians are encouraged to use their judgement in accordance with clinical guidelines. Clinicians may refer to the trial medication guide for ASCEnD (online supplemental appendix 7) which contextualises NICE guidelines^13^,^15^ and the British National Formulary (BNF) dose schedules^84 93 111^ in relation to study-specific considerations. For instance, the guide suggests maximum doses for aripiprazole (10 mg/day), quetiapine (300 mg/day) and sertraline (150 mg/day) that are lower than the maximum BNF doses.^84 93 111^ Prior to initiating trial medication, adjustment or discontinuation and washout of existing medication may be required at the discretion of the prescribing clinician.

Decisions regarding dosage and the ongoing use of trial medication after the 24-week follow-up remain the responsibility of the prescriber and may be informed by a participant’s self-reported questionnaire scores and feedback on mood symptoms from cRAs. Clinical teams are free to adjust all other medication per local clinical procedures and may reference the BNF^84 93 111^ to review cautions and contraindications.

### Outcomes

#### Baseline

Participants complete primary and secondary baseline measures at randomisation. Informal carers complete baseline measures within 2 weeks of the corresponding participant.

#### Primary outcome

The effectiveness of aripiprazole/sertraline combination in improving depressive symptoms is assessed using the QIDS-SR 12–16 weeks after randomisation.

#### Secondary outcomes

The impact of trial medication on the following is measured:

- Trajectories of symptom change using the QIDS-SR^107^ reported weekly from screening.
- Treatment acceptability using the Treatment Satisfaction Questionnaire for Medication^112^ at baseline and weeks 4, 14 and 24.
- Tolerability using the Glasgow Antipsychotic Side-effect Scale (GASS)^113^ at baseline and weeks 4, 14 and 24.
- Pattern of medication adherence from data collected during study calls on study medications taken on each of the preceding 7 days, supported by use of the Medication Adherence Rating Scale^114^ at baseline and weeks 4, 14 and 24.
- Pattern of non-randomised antipsychotic/antidepressant medication use from concomitant medication data collected during weekly calls.
- Change in anxiety symptoms using the Generalised Anxiety Disorder-7^115^ scale weekly from baseline.
- Change in manic symptoms and rates of relapse to a hypomanic or manic episode using the Altman Self-Rating Mania Scale^116^ weekly from baseline.
- Psychosocial functioning using the Work and Social Adjustment Scale^117^ at baseline and weeks 4, 14 and 24.
- Health-related quality of life using the EQ-5D-5L^118^ questionnaire at baseline and weeks 4, 14 and 24.
- Capability well-being using the ICEpop CAPability measure for Adults (ICECAP-A)^119^ and the Oxford CAPabilities Questionnaire-Mental Health (OxCAP-MH)^120^ at baseline and weeks 4, 14 and 24.
- Costs and incremental cost-effectiveness using the Health Economics Questionnaire,^108 121^ measured at baseline and weeks 4, 14 and 24.
- Informal carers’ health-related quality of life, capability well-being and costs of caring using the EQ-5D-5L, ICECAP-A, OxCAP-MH and Caregiver Indirect and Informal Care Cost Assessment Questionnaire (CIIQ)^122^ at baseline and weeks 4, 14 and 24.

#### Exploratory outcome

Outcome responsiveness is measured using the Probabilistic Instrumental Learning Task (PILT),^123^ and the Behavioural Inhibition System/Behavioural Approach System (BIS/BAS)^124^ scales. Reward responsiveness is assessed through choice accuracy in win trials of the PILT and the total score on the BAS Reward Responsiveness subscale of the BIS/BAS. Punishment responsiveness is evaluated through choice accuracy in loss trials of the PILT and the total score on the BIS subscale of the BIS/BAS. Baseline measures are completed after randomisation, prior to trial medication initiation. Follow-up measures are taken 2–4 weeks and 12–14 weeks after trial medication initiation. Participants who commenced treatment of aripiprazole or quetiapine prior to the trial and are subsequently randomised to the same arm do not complete exploratory measures.

### Harms

Adverse events are recorded from randomisation to the end of participation. The GASS^113^ is used to collect a subset of side effects which are not reviewed for severity or causality, given the trial medications have marketing authorisation and extensive safety data is available.^84 93 111^ Serious adverse events and suspected adverse reactions are identified by research staff during study follow-up and reported in accordance with Medicines and Healthcare products Regulatory Agency (MHRA) and Research Ethics Committee (REC) requirements.

Early identification and appropriate management of potential risks to participants throughout the trial are managed by weekly cRA safety monitoring calls and alerts from the study database. During calls, risks such as safeguarding concerns, pregnancy, escalating suicidal ideation and/or deterioration in mood and affective symptoms are screened. If identified, cRAs notify healthcare professionals who hold clinical responsibility for the participant. Automated email alerts regarding possible domestic abuse or suicidal intent are flagged from participant responses to self-reported questionnaires and sent to their local research team and cRAs for follow-up.

### Participant pathway

**Figure 1:**
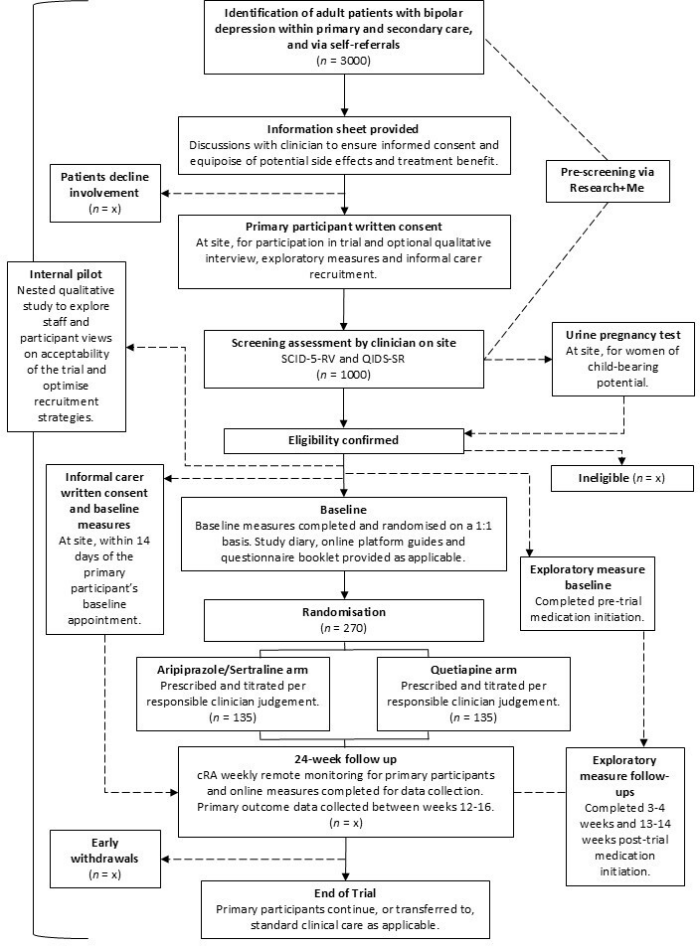
Participant pathway. cRAs, central Research Assistants; QIDS-SR, Quick Inventory of Depressive Symptomatology—Self Report; SCID-5-RV, Structured Clinical Interview for Diagnostic and Statistical Manual-5-Text Revision Disorders-Research Version.

The participant pathway is shown in figure 1.

### Data collection

Primary and secondary outcome measures are completed by participants and informal carers online using the electronic patient-reported outcomes system and an instruction guide for the system is provided as required (online supplemental appendix 8). For participants where online completion is not possible, a questionnaire booklet (online supplemental appendix 9) is provided for reference during remote support from research staff. Exploratory measures are completed by participants on the Gorilla Experiment Builder^125^ platform, supported by a participant instruction guide for the platform (online supplemental appendix 10).

Participants are provided study diaries (online supplemental appendix 11) at the start of randomisation to record the dose of trial medication taken each day (including missed and incorrect doses) and any changes to concomitant medication. cRAs contact participants weekly via telephone or videoconference during the 24-week follow-up period to collect data on trial medication doses and concomitant use. They additionally monitor, prompt and assist participants with questionnaire completion and exploratory measures as required. Weekly calls facilitate safety monitoring as described under Harms.

Participants are recompensed when they complete baseline, week 14 and week 24 assessments. Participants continue participation in the trial if they choose not to commence trial medication or discontinue trial medication during the follow-up period. Withdrawn participants are not replaced.

### Data management

Data is managed, computerised and stored in accordance with the^126^ Good Clinical Practice (2005/28/EC) and local policy. The overall quality and completeness of trial data is the responsibility of the chief investigator (CI). cRAs receive participant identifiable information to enable implementation of trial monitoring and risk procedures. Study data is entered and recorded on the trial data management system, Red Pill, using unique trial identifiers assigned to participants and informal carers at recruitment. Exploratory measures are completed by participants on the Gorilla Experiment Builder^125^ platform and stored using their trial identifiers.

### Data analysis

The analysis will follow a detailed prespecified statistical analysis plan (SAP) that will be finalised and signed off before the database is locked and any trial data is accessed. The primary analysis population is the intention-to-treat (ITT) population, that is, participants analysed according to randomisation allocation. The analysis will compare all weekly QIDS-SR scores^107^ available between 12–16 weeks in the two arms, adjusting for baseline QIDS-SR score (taken at screening) and stratification factors. Mixed effects linear regression will be used to account for repeated measurements and the model will be fitted to all available QIDS-SR data. One supplementary analysis will adjust for the stratification factors as in the ITT analysis but will be conducted on the per-protocol population (efficacy population) which will be further defined in the SAP. A sensitivity analysis will repeat the primary analysis with the inclusion of outcomes for all randomised participants using appropriate multiple imputation techniques to infer values for missing data.

The primary outcome assessment period, 12–16 weeks, is used because data from other studies of quetiapine in bipolar depression^17 90 91^ and bipolar disorder,^127^ and of sertraline^128 129^ and aripiprazole^59 60^ in unipolar depression studies, show that the point of maximum separation occurs 6–8 weeks after dose escalation. The 12–16 week period allows time for transition to the trial medication and for response to be optimised in both treatment arms. 12 weeks is also the time point used for assessment of acute efficacy in previous CEQUEL^92^ and PAX-BD^130 131^ studies in bipolar depression. Analysis of secondary outcome measures allows examination of trajectories of symptom change^107 115 116^ and the pattern and cause of drug discontinuation over 24 weeks.^112^,^114117^

#### Health economic analysis

A prospective, within-trial economic evaluation will be conducted primarily from the health and social care perspective with secondary analyses incorporating wider societal costs (eg, lost productivity, informal care) for the ITT sample over 24 weeks and will closely follow the methods of the CEQUEL^94^ and the PAX-BD economic evaluations.^130 131^ Costs will be calculated by multiplying collected resource use information with matching UK national-level unit costs from routine sources.^132^ Quality-adjusted life years derived from the EQ-5D-5L^118^ will be assessed as the main outcome. With concerns about the responsiveness and comprehensiveness of the EQ-5D-5L in bipolar disorder,^133^ alternative capability well-being outcomes (OxCAP-MH,^120^ ICECAP-A^119^) and derived capability-weighted life years will also be measured. Missing cost and outcome data will be handled using multiple imputation methods. Differences between the two arms will be investigated within an incremental cost-effectiveness/cost-utility framework with extensive uncertainty and sensitivity analyses including per-protocol and complete case samples.

In addition, the effects on informal carers’ health-related quality of life, capability well-being and their time and costs (based on the EQ-5D-5L, ICECAP-A, OxCAP-MH and the CIIQ^122^ completed by informal carers at baseline and weeks 4, 14 and 24) will be analysed and taken into consideration together with data from participants to investigate the cost-effectiveness jointly for the service user/informal carer dyad.

#### Equity analysis

The impact of socioeconomic status, age, gender and ethnicity (characteristics identified by the PROGRESS framework^134^ as being important to consider in terms of equity of inclusion in health research) on recruitment of participants, participation and the clinical and cost-effectiveness of the study treatments will be determined. This will be examined by comparing these characteristics in populations eligible for and engaged in the trial at different points in the study pathway. Relevant populations include the screening and randomisation populations for each study site and individuals who click on social media advertisements organised by Just-R, register with Research+Me, are invited to complete the initial eligibility assessment, complete the initial eligibility assessment, appear eligible following the initial eligibility assessment, successfully contact sites to arrange a screening appointment, consent to participate, remain in the study until the primary outcome point and complete the study. All demographic details received from individuals within the Research+Me system are anonymous. Where possible and with due accord to clinical governance pertaining to identifiable data, Clinical Practice Research Datalink,^135^ Primary Care Networks and individual primary care centre records will be utilised to examine the impact of deprivation and ethnicity on primary care recruitment.

#### Exploratory analysis

The trial medication initiation date defines day 0 for the exploratory outcome. Exploratory analyses will examine reward processing changes from baseline (pre-trial medication initiation) to 14 weeks post-trial medication initiation and their association with trajectories of symptom change over the full 24 weeks. These analyses will follow a fully detailed prespecified SAP reviewed by the trial oversight committees.

### Nested qualitative study

The feasibility and acceptability of the study is examined through 25 participant and 30 clinician perspectives on recruitment procedures, trial intervention, reasons for withdrawal, diagnosis and management of bipolar disorder and challenges associated with accessing or delivering specialist care within current NHS systems and changes in clinician workload. Interviews are semi-structured and conducted via telephone or virtual platforms; recordings are transcribed verbatim for analysis, anonymised and stored securely for up to 5 years. Thematic analysis^136^ is conducted within the datasets, followed by a framework analysis based on the Theoretical Framework of Acceptability^137^ across the datasets. The findings are used to inform refinement of recruitment procedures and other study processes. The protocol for the nested qualitative study has been published.^138^

### Monitoring

The Data Monitoring Committee (DMC) is responsible for reviewing all trial safety data. The DMC consists of an independent chair, an experienced independent statistician and at least one independent psychiatrist with an interest in bipolar disorder. There are no formal interim analyses planned except for snapshots reported to the DMC, and therefore no criteria for the premature termination of the trial. They meet at least annually throughout the trial and make recommendations to the Trial Steering Committee (TSC) regarding trial progress. The TSC has the authority to recommend changes to the protocol and/or ongoing progress of the study. The TSC consists of an independent chair with at least two other independent members, an informal carer representative, the CI and deputy CI. Membership of both oversight committees has been agreed by the funder. The trial may be subject to audit by sponsor representatives or inspection by the MHRA or NIHR Health Technology Assessment. Each investigator site permits trial-related monitoring, audits and regulatory inspection including access to all essential and source data relating to the trial.

### Patient and public involvement statement

The application for funding and ethical approval of the ASCEnD trial was informed by lived experience and the protocol was designed with significant input from the Lived Experience Advisory Panel (LEAP), supported by the McPin Foundation. LEAP members provide feedback on an ongoing quarterly basis regarding study progress and inform trial oversight committee decisions. Recruitment of trial participants and dissemination of results are supported by the McPin Foundation. The panel has also been involved in recruiting and training participant-facing cRAs; this has supported staff development, participant engagement and retention by enhancing the quality and acceptability of weekly follow-up calls comprising discussion of sensitive topics. Qualitative analysis is conducted in collaboration with the LEAP to broaden insights, interpretation and application of findings.

## Ethics and dissemination

###  Ethical approval

The trial is conducted in accordance with the Medicines for Human Use (Clinical Trials) Regulations 2004 and in accordance with the principles of the Declaration of Helsinki. A Clinical Trial Authorisation from the MHRA, approval from the Health Research Authority (IRAS 1007468) and a favourable opinion from North East – Newcastle and North Tyneside 1 REC (23/NE/0132) was obtained prior to the start of the study. Investigators, RECs, trial participants and trial registries are notified of protocol amendments as appropriate.

### Consent

Clinicians are encouraged to make a collaborative decision with service users regarding the equipoise of potential side effects and treatment benefit. Written informed consent is provided to trial clinicians using the Participant Consent Form (online supplemental appendix 12) prior to any study related activities. Participants may optionally consent to completing exploratory measures, being contacted for qualitative interview and informal carer recruitment. Informal carer written informed consent is obtained by trained site staff using the Informal Carer Consent Form (online supplemental appendix 13). Participants and informal carers have the right to withdraw consent from the study at any time without providing a reason. Consent for publication is not applicable.

### Confidentiality

The study is run in accordance with the Data Protection Act 2018^126^ and covered by a Data Protection Impact Assessment to safeguard the confidentiality of participants and informal carers. Non-identifiable research data may be shared with researchers in other universities and organisations, including those based outside the UK, for research in health and social care. A final trial de-identified dataset will be prepared and stored by Newcastle University. Any sharing of identifiable information requires explicit participant consent and appropriate safeguards are in place for international data transfer.

### Post-trial care

After 24 weeks of follow-up, participants end the trial and those seen in research clinics continue in, or are transferred to, standard clinical pathways. The study end does not prompt change in ongoing drug treatment, including stopping trial medication, as this is prescribed and dispensed locally at sites.

### Dissemination policy

Dissemination will include a final report to the NIHR. Findings will be disseminated to the academic and clinical community via conference presentation and publication in peer-review journals. The study LEAP will be acknowledged in all study publications. A plain English ‘lay’ summary of the findings will be disseminated to participants via social media and presentation at Bipolar UK meetings. A graphical summary of the results will also be produced by the McPin Foundation and be made publicly available.

## Discussion

This trial addresses a key evidence-practice gap in the treatment of bipolar depression by comparing an antipsychotic/antidepressant combination with quetiapine, an established monotherapy. The use of widely prescribed, cost-effective trial medications supports the pragmatic design, but open-label prescribing could influence participant-reported outcomes across the treatment arms. If the sample target is met via the broad recruitment pathways, this may strengthen the applicability of findings to routine clinical practice.

## Status of the study

Recruitment for the ASCEnD trial was originally intended to commence in June 2023 and end in August 2025. However, due to unavoidable delays, the study began in April 2024 with a current planned recruitment end date of August 2027.

## Supplementary material

10.1136/bmjopen-2025-112677 online supplemental material 1

10.1136/bmjopen-2025-112677 online supplemental appendix 1

10.1136/bmjopen-2025-112677 online supplemental appendix 2

10.1136/bmjopen-2025-112677 online supplemental appendix 3

10.1136/bmjopen-2025-112677 online supplemental appendix 4

10.1136/bmjopen-2025-112677 online supplemental appendix 5

10.1136/bmjopen-2025-112677 online supplemental appendix 6

10.1136/bmjopen-2025-112677 online supplemental appendix 7

10.1136/bmjopen-2025-112677 online supplemental appendix 8

10.1136/bmjopen-2025-112677 online supplemental appendix 9

10.1136/bmjopen-2025-112677 online supplemental appendix 10

10.1136/bmjopen-2025-112677 online supplemental appendix 11

10.1136/bmjopen-2025-112677 online supplemental appendix 12

10.1136/bmjopen-2025-112677 online supplemental appendix 13
