# Supplementary material for: Open-label randomised controlled trial of aripiprazole/sertraline combination in comparison with quetiapine for the clinical and cost-effectiveness of treatment of bipolar depression (the ASCEnD study): study protocol
Source: BMJ Open. 2026 Mar 19;16(3):e112677. doi: 10.1136/bmjopen-2025-112677 (PMC13007169; doi:10.1136/bmjopen-2025-112677)
Supplement: online supplemental appendix 1 [file bmjopen-16-3-s002.pdf]

Dear [** **], We at the [GP practice name] are supporting a new, important research study into mental health that you may be interested in taking part in. For more information, please visit the The ASCEnD Trial website. Dr. [** **]
