# Supplementary material for: Open-label randomised controlled trial of aripiprazole/sertraline combination in comparison with quetiapine for the clinical and cost-effectiveness of treatment of bipolar depression (the ASCEnD study): study protocol
Source: BMJ Open. 2026 Mar 19;16(3):e112677. doi: 10.1136/bmjopen-2025-112677 (PMC13007169; doi:10.1136/bmjopen-2025-112677)
Supplement: online supplemental appendix 2 [file bmjopen-16-3-s003.pdf]

<<GP practice logo>>

<<Date>>

Dear <<Title>> <<First Name>> <<Surname>>,

Invitation to participate in research study

[GP practice name] is supporting a new research study into mental health that you may be interested in taking part in.

Why take part in research?
Research helps to improve the care provided by the NHS. It's the driving force behind new advances in medicine, helping find new cures and better treatments for all. But it's the people who take part in health research who make these vital discoveries possible.

What is the Study?
The research study is called **ASCEnD** and it is a clinical trial looking at the effectiveness and usefulness of using the drugs sertraline and aripiprazole together in comparison to using quetiapine alone as treatments for people living with bipolar and who are currently experiencing depression. All of these drugs are already commonly used in the NHS.

Might I have bipolar? [Delete this paragraph if sending this letter/email to those who already have a diagnosis of Bipolar]
Receiving this study information does not mean you have been identified by us as having bipolar, or that we suspect you may have bipolar. Symptoms of bipolar can include not feeling like your usual self, experiencing racing thoughts and being unable to slow your mind down. For more information about biopolar (either for you or your family members or carers), visit the BipolarUK charity website: www.bipolaruk.org/diagnosing-bipolar#could-it-be-bipolar

If you think the study might be suitable for you and would like to know more about it, please read the patient information sheet enclosed with this letter. For more information, you can also visit the **ASCEnD** Trial website: www.ascendtrial.co.uk/patient-homepage

If you have any questions about the study or not sure if the study is suitable for you, please feel free to contact the GP practice team on [INSERT GP number] or your local ASCEnD study team directly on [INSERT site team/CRN number as appropriate] who will be happy to speak with you. Your local ASCEnD study team is part of [Insert Trust name here].

Via the trial website, you can register your interest in taking part in the trial. Alternatively, you can contact us at the GP practice, and we can organise a referral to your local study team, on your behalf.

REMOVE IF NOT APPLICABLE You may receive a phone call from [GP practice team/LCRN team] following receipt of this letter to discuss this trial. If you don't want to speak about it, please just let us know if we call.

Thank you for reading this letter.
<Lead GP signature and name>
