# Supplementary material for: Open-label randomised controlled trial of aripiprazole/sertraline combination in comparison with quetiapine for the clinical and cost-effectiveness of treatment of bipolar depression (the ASCEnD study): study protocol
Source: BMJ Open. 2026 Mar 19;16(3):e112677. doi: 10.1136/bmjopen-2025-112677 (PMC13007169; doi:10.1136/bmjopen-2025-112677)
Supplement: online supplemental appendix 3 [file bmjopen-16-3-s004.pdf]

Email to Be Part of Research volunteers

This document details the required text (in black) for the email that the Be Part of Research team will send to volunteers on behalf of the study. Please provide the information indicated by the red text and refer to the yellow highlighted notes for further instructions.

To: <volunteer>
From: Be Part of Research <bepartofresearch@nihr.ac.uk>
Subject: New study match on Be Part of Research

NIHR/Be Part of Research Logo (supplied by NIHR)

Dear <name>

You have received this email because you are registered with Be Part of Research and have indicated that you are interested in hearing about new research opportunities.

Study summary

"ASCEnD" is a clinical trial for people who have bipolar and are currently experiencing depression.

There are currently limited treatment options available for people with bipolar who have depressive symptoms. The ASCEnD trial will examine whether using two treatments together is better than standard single-drug treatment for bipolar. All of these drugs are already used in the NHS, and their safety profile is well known.

Who can take part?

You may be able to take part if you:

- Have bipolar
- Are currently depressed
- Are over the age of 18
- Not currently pregnant or breastfeeding or planning to become pregnant during the trial

Might I have bipolar?

Has there ever been a period when you were not your usual self and thoughts raced through your head or you couldn't slow your mind down? If so, you will find more information on the BipolarUK website: www.bipolaruk.org/diagnosing-bipolar#could-it-be-bipolar.

What does the study involve?

Taking part will involve attending one or two face-to-face appointments (most likely one), to learn more about the trial and help you decide whether or not to take part, as well as confirming if you are eligible to take part. This will include completing some questionnaires, and discussing your medical history and medication.

If you agree to take part and are eligible, you will be randomly selected by a computer to start one of the trial treatments. If the treatment suits you and seems to be working then you will be able to continue it. If it isn't, then you don't need to, and you and your doctor can change to something else and you can still take part in the trial.

During the trial, you will be asked to complete a number of online questionnaires on a weekly basis, and you will receive a weekly phone call from a member of the research team for support.

You will be asked to take part in the trial for 24 weeks. You will be given up to £50 in vouchers, as a thank you for giving your time for the trial. You will be also reimbursed for reasonable travel costs for the in-person trial appointment(s).

Where can I take part?

The study is taking place at multiple sites across 10 Mental Health Trusts in England.

For more information

Please visit the study website: www.ascendtrial.co.uk/patient-homepage where you can get further information, register your interest for the trial and find out if you may be eligible to take part.

This study is being run with the support of the NHS and the National Institute for Health and Care Research (NIHR), working in partnership with Cumbria, Northumberland, Tyne and Wear NHS Foundation Trust and Newcastle University.

As a Be Part of Research volunteer, you may have received an invitation to join this or another study before. If you are currently taking part in another study, please let the study team know when they contact you.

If you have any questions about the study itself, please contact the study team directly using the contact method above. You don't need to let us know if this study is not suitable for you. If you'd like to search for other research opportunities, you can do so on the Be Part of Research website. Thank you for your ongoing support of Be Part of Research. If you'd like to receive all our latest news and hear of more opportunities to take part in health and care research from across the UK, you can sign up to our newsletter. If you no longer wish to be part of this service, you can withdraw your permission at any time. Please visit the close your account page for more information.
