# Supplementary material for: Open-label randomised controlled trial of aripiprazole/sertraline combination in comparison with quetiapine for the clinical and cost-effectiveness of treatment of bipolar depression (the ASCEnD study): study protocol
Source: BMJ Open. 2026 Mar 19;16(3):e112677. doi: 10.1136/bmjopen-2025-112677 (PMC13007169; doi:10.1136/bmjopen-2025-112677)
Supplement: online supplemental appendix 5 [file bmjopen-16-3-s006.pdf]

Aripiprazole Sertraline Combination Effectiveness

Aripiprazole/**S**ertraline **c**ombination: clinical and cost-**e**ffectiveness in comparison with Quetiapine for the treatment of bipolar **d**epression. An open label randomised controlled trial.

The ASCEnD Study

Participant Information Sheet

INVITATION

You have been given this information sheet because you **may** be eligible (suitable) to take part in a research study called ASCEnD.

Taking part in ASCEnD is voluntary but before you decide whether to take part, it is important that you understand why the study is being done and what it would involve for you.

Please read the following information to help you decide if you would like to take part. You may wish to discuss it with friends or family first. If you decide not to take part, this will not affect the care that you receive. If anything is unclear, or you need more information, please ask the research team using the details at the end of this sheet.

Thank you for taking the time to consider being part of the ASCEnD study.

STUDY SUMMARY

- You have been invited to take part in the ASCEnD study as your clinical care team think you could be eligible, or you may have received an invitation from your GP or a research registry, or heard about the study from social media or word of mouth.
- The study is being done to test the effectiveness and usefulness of the drugs sertraline with aripiprazole or quetiapine as treatments for people living with bipolar who are currently experiencing depression. All of these drugs are currently used in the NHS.
- If you agree to take part, you will be involved in the study for up to 24 weeks.
- You will be randomly selected, by a computer, to take either a combination of sertraline and aripiprazole (“sertraline/aripiprazole combination”) **or** quetiapine. Your study doctor will work with you to help you take part in the study. There is an equal (or 50%) chance of you being randomly selected to either option. You and your clinical team will know whether you have been randomly selected to take a sertraline/aripiprazole combination or quetiapine.
- You will be asked to attend an appointment with the research team at your local hospital, local research centre, or with your community mental health team to:
    - ✓ provide written consent to say you agree to take part
    - ✓ confirm if you are eligible to take part
    - ✓ be randomly selected to receive sertraline/aripiprazole combination or quetiapine
    - ✓ complete some questionnaires about you and your health.
- You will be asked to complete weekly online questionnaires at home. They will ask you about your mood and quality of life throughout the study.
- Study research assistants working with Cumbria, Northumberland, Tyne and Wear NHS Foundation Trust will contact you (e.g. by telephone or videocall) each week throughout the study. They will ask you about your mood, behaviours, symptoms and medication. They can also help you complete the study questionnaires.
- If you have a carer (e.g. a friend, family member or someone else who supports you without being paid), we would also like them to be part of this study, but this is entirely optional.

If you are interested in taking part, please read on for further details.

What is the ASCEnD Study?

Bipolar disorder (referred to as ‘bipolar’ in this document) occurs in about 1.4% of people, over a lifetime. There are currently limited treatment options available for people experiencing depressive symptoms. In addition, some of these drugs have side effects such as sedation (feeling very sleepy) and gaining weight, and often cannot be prescribed by a GP.

Although antidepressants can be very effective in people with depression and can also be prescribed by a GP, very little is known about using antidepressants in patients living with bipolar. In this study, we will use a combination of an antidepressant (sertraline) and an antipsychotic drug (aripiprazole) and compare this with an antipsychotic drug called quetiapine. Quetiapine is a common treatment for people experiencing depression in bipolar. All three drugs are already used in the NHS, but we want to find out if using a sertraline/aripiprazole combination will be beneficial in reducing depressive symptoms in people with bipolar.

To do this, we are looking for 270 patients with bipolar who are currently experiencing depression from across the UK to take part in the ASCEnD study.

Who can take part in ASCEnD?

If you have a diagnosis of bipolar, are aged 18 or over and are currently experiencing depression, you may be able to take part. We are not able to include anyone in the trial who is pregnant, planning to become pregnant during the study period (6 months) or currently breastfeeding, or anyone with a severe substance use disorder.

Do I have to take part?

No. You can decide if you want to take part. If you choose not to, you will continue to take your standard treatment as arranged by your usual clinical care team.

If you agree to take part, you can change your mind and withdraw from the study at any time without needing to give a reason. Your care will not be affected. However, **it is important that you do not suddenly stop taking your medications.** Your usual clinical care team will help you manage your medications.

If during the study new information becomes available that is relevant to you, we will tell you about it as soon as possible. We will discuss whether you would like to withdraw as a result.

STUDY PARTICIPATION SUMMARY

Appointment to find out more and to check if you want to and can take part

Initial Study Appointment

- Opportunity for you to discuss the study with a member of the research team
- **Sign a consent form**
- Complete questionnaires and answer some questions (this will take about 60 minutes) to find out if you are able to take part

Baseline *(may be at the same appointment as the initial appointment)*

- Complete study questionnaires (this may take about 1 hour)
- The study team will collect relevant study information about you including your current medications, medical history (time since your bipolar diagnosis) and demographic details (including your initials, age, sex, gender, postcode, highest level of education, family status and ethnicity).

Take part in the study for 24 weeks

Study Medication

- If you are eligible to take part and once you have completed the baseline questionnaires, you will be randomly selected to take either a combination of sertraline and aripiprazole, or quetiapine.

Group 1

Sertraline/Aripiprazole combination

Group 2

Quetiapine

Follow the treatment plan given to you by your doctor

Questionnaires
(weeks 1 to 24)

- Complete weekly questionnaires
- At 4 weeks, 14 weeks and 24 weeks following your baseline visit, you will have additional questionnaires to complete
- ***Optional:*** REWARD study tasks
- Reminders will be sent to your phone or email

Calls with Research Assistants

- Regular weekly calls with study research assistants.

Optional: Interview with a research team from Keele University.

End of Study Participation

What would taking part involve?

Initial Study Appointment
A member of the ASCEnD study team will discuss the study with you and check whether you are able to take part. This will be done at a face-to-face appointment, which the study team will arrange with you. At your initial appointment, a study doctor will:

1. Discuss the study with you and answer any questions you may have
2. Ask you to complete and sign a consent form. You will receive a copy of this.
3. Do some checks to confirm that you can take part in the study. This will involve looking through your medical history and asking you some questions. It may also include asking you to complete a questionnaire called the Quick Inventory of Depressive Symptomatology – Self Report (QIDS-SR) and doing the Structured Clinical Interview for DSM-5 Disorders – Research Version (SCID-5-RV), if you haven't completed these recently. This will take about 60 minutes to complete.

The study doctor will then confirm whether you can take part in the study. If you are not able to take part you will continue under the care of your usual clinical care team. If you are able to take part, you will be asked to complete some more questionnaires, known as the baseline questionnaires, which will tell us about your quality of life. This might be at the same appointment, or at a separate appointment if you prefer. You will then be randomly selected to take either:

A) Sertraline/Aripiprazole combination or
B) Quetiapine

You will have equal chance of being in group A) or group B). Which group you are in will be selected by a computer.

Taking study medication
At your initial appointment, you will be given a study diary. We would like you to use this throughout your time in the study to write down when you start taking your study medication and to record how much you take on each day. There will also be space for you to write about any other medications you may be taking.

Your study medication may be prescribed and managed by the study doctor at the hospital where you attend your study appointment, or it may be done by your usual clinical care team. You will need to collect your prescription in the same way as you would if you weren't in the study.

To start the study medication, you may need to stop taking some medications that you are currently taking. Your doctor will talk to you about the best way to do this. Please make sure that you follow their advice.

Completing questionnaires
We would like you to complete questionnaires each week for 24 weeks, which will help us understand whether the study medications are helpful in treating bipolar depression. You will have additional questionnaires to complete on weeks 4, 14 and 24 (with week 1 starting after your initial study appointment).

After giving your consent to take part in the study, you will be given access to an online system called ePRO. You will use ePRO to complete your questionnaires, and will receive reminders via email and text message to complete them. If you would prefer, you can instead complete your questionnaires over the phone or in a video call with a study research assistant or your local study team.

Speaking with a study research assistant
After your appointment, a research assistant will contact you via telephone or video call to introduce themselves and to see when is best for you to talk weekly for up to 24 weeks. During these calls, the research assistant will ask you about:

- how you are feeling.
- whether you are experiencing any side-effects.
- if you might be pregnant (if applicable).
- the information in your study diary.
- how you are getting on with completing the study questionnaires. If you find completing the study questionnaires to be difficult or upsetting in any way, the study research assistants will provide help and if needed signpost you to appropriate sources of support.

Please note that the study research assistants are not medically qualified and can't advise you on your medication or on any symptoms. You should instead discuss any concerns you have with your usual clinical care team.

Travel Expenses and Payment
Reasonable travel expenses for your initial study appointment(s) will be reimbursed, please remember to keep receipts to enable this to happen. We will also offer you a further £50 total (£15 following your first appointment, £20 at week 14 and £15 at the end of the study) as a thank you for all of your time and effort. This will most often be in the form of gift vouchers, but some local study teams may be able to offer payment to bank accounts (you are advised to check if this will impact on your benefits).

Optional Interview
We would like to find out about your views and experiences of being involved in ASCEnD and how mental health services could be improved. To do this, we may invite you to take part in an interview which is being organised by part of the research team, who work for Keele University, if you consent to this. **The interview is optional, and you do not have to take part in this.** We will only carry out a certain number of interviews and therefore not everyone who agrees to this optional interview will be contacted. The interview may be conducted by telephone, video call or if you prefer a face to face interview, this may be accommodated in line with COVID-19 guidance.

If we randomly select you to take part in an interview, a member of the study team will contact you to arrange a convenient date and time to do the interview. At the start of the interview, we will ask you to provide verbal consent to continue with the interview and we will send you a copy of this consent form by post or email. The interview will last approximately 45 minutes and will be recorded. After the interview has taken place, we will make a transcript, which means typing out what you have said. Your name and other personal details will not be included on the transcript and you will not be given a copy to keep. Some of your transcript may be quoted in scientific papers, but this will not be linked back to you in any way. You will be reimbursed with a £25 gift voucher for your help with the interview.

Optional REWARD Study

We are also interested in looking at how the study medication might affect emotion and behaviour (ability to experience pleasure and learn from positive outcomes), so that we can learn more about how these medications work and help to improve quality of life. We will be looking at this using a computerised decision making-task and questionnaire using a website designed for collecting information called “Gorilla”.

If you decide you are interested, consent to take part will depend on which medicines you are currently taking and you might not be able to take part if you already take one of the study medications. You must also be able to complete the initial REWARD study tasks before starting to take your study medication.

To take part in this optional part of the study you will need access to a computer, tablet, or smartphone with an internet connection, as unfortunately we are unable to provide these tasks over the phone. You will be given an online login and instructions on how to do this. This is a separate login to the weekly ePRO questionnaires you will complete as part of the main study. We will send you reminders to complete REWARD tasks via text message/email and the weekly cRA call, if you decide to take part. **The involvement in this part of the study is optional and you do not have to agree to this.**

You will then be asked to complete the same assessments twice more (around 3 weeks and 13 weeks after you start taking medication). Each sitting will take a maximum of 45 minutes to complete all the tasks (which includes optional breaks). This may take less time.

Optional questionnaires for carers

You might have a partner, family member or friend who supports and helps you with activities without being paid which you may be referred to as your ‘carer’. If they are aged over 18 years old, we would like to ask them to be part of the study too. We will only approach your carer to be part of the study with your consent. **The involvement of your carer in the study is optional, and you do not have to agree to this.**

If your carer would like to take part, they will be asked to sign a consent form. Taking part will involve completing some questionnaires around the time of your study appointment, and after 4, 14 and 24 weeks. They will be provided with their own log in details to complete online questionnaires on ePRO. We will not share any of your information with them and we will not share any of their answers with you.

Study end
You will continue to receive care from your usual clinical care team and will not be followed up by the research team. This may include continuing to take the medication you have been prescribed during the study if you feel it was helpful. It is important that you follow the advice given by your clinical care team doctor once you reach the end of the study.

What are the possible benefits and risks of taking part?

As part of the study, you will speak regularly with a study research assistant. You may find this a helpful source of support.

We think the risks to being part of the study are low as all of the study medications are currently used in the NHS. However, all medications carry some risks. Some of the common side effects of these drugs include:

- Anxiety, nausea and dizziness (sertraline)
- Dizziness, drowsiness and weight gain (aripiprazole)
- Dizziness, drowsiness, increased sleep and weight gain (quetiapine)

Your study doctor will discuss all of the possible risks and benefits of these medications with you. **If you experience any side effects while taking any of the study medications, it is important that you tell your clinical care team straight away**.

What if I'm pregnant or planning to become pregnant?

You are not able to take part in the study if you are pregnant, breastfeeding or planning to become pregnant during the time you would take part in the study (6 months). If relevant, the study doctor will discuss the possibility of you becoming pregnant over the course of the trial and this discussion may include questions about contraception. If you are a woman, sexually active, not using highly effective contraception and not post-menopausal, you will be required to have a pregnancy test before starting the trial. The study medications have potential risks in pregnancy and your study doctor will need to be confident that you understand these risks.

We will ask you through the trial about possible pregnancy- e.g., we will ask you to tell us if you are pregnant, planning to become pregnant, or have changed your contraceptive practice. If you become pregnant, we will ask you as part of the consent process to confirm you are happy for the research team to follow your pregnancy until the outcome is known (for example, until you have your baby). This will include regular telephone calls or video calls with you to check how you are feeling and to check if you are experiencing any issues.

What happens if I decide I don't want to continue with the study?

If you would like to stop taking part, we would like you to talk to your study doctor. You are free to discontinue your involvement in the study at any time, but any decision you have about your treatment needs to be discussed with your treating doctor. If you stop taking study medication or change medications, it is still very valuable, but not compulsory, if you continued to complete the questionnaires and phone calls.

If your study doctor thinks it would be beneficial for you to stop taking part in the study, or you lose your mental capacity to continue with the study, you will be withdrawn from any further involvement.

If you no longer wish to take part in the optional REWARD study or optional interviews, please just let the research assistants know when they call, or contact your local study team using the details at the end of this leaflet.

Please also see page 12 for "Your rights with regards to your data".

Will my GP know that I'm taking part in the study?

Yes, your local study team will send a letter to your GP to inform them that you are taking part in this study. A copy of this letter will also be filed in your medical notes. This is so your medical records at your GP practice and at the hospital contain documentation confirming that you are taking part in this research.

Is there anything else I need to know?

Organisation and funding of the study
Chief Investigator: The doctor in charge of the study is Dr Stuart Watson, a Consultant Psychiatrist. He works for Newcastle University and at Cumbria, Northumberland, Tyne and Wear NHS Foundation Trust.

Study Sponsor: Cumbria, Northumberland, Tyne and Wear NHS Foundation Trust is the sponsor of the study. This means they have overall responsibility. The study is managed by the Newcastle Clinical Trials Unit, a part of Newcastle University, on behalf of the Sponsor. Researchers from Keele University are organising and conducting the optional interviews.

Study Funders: The study has received funding as part of the National Institute for Health Research (NIHR) Health Technology Assessment (HTA) programme (reference NIHR132773). The NIHR is funded by the UK Government to carry out research for the benefit of the NHS and its patients.

A mental health research charity called the McPin Foundation (www.mcpin.org) is supporting the involvement of people with lived experience of bipolar in this study.

Ten NHS Hospital Trusts will be taking part in this study. Each trust will have a lead study doctor, called a Principal Investigator. The Principal Investigator at your NHS Trust is _____________ (to remove for online generic version)

Review of the study
The study has been reviewed by independent experts from the NIHR. They ensure the study is an accurate way to understand whether an aripiprazole/sertraline combination may be beneficial to individuals experiencing bipolar depression.

The study has also been reviewed by, and received approval from, North East- Newcastle and North Tyneside Research Ethics Committee 1 (REC Reference: 23/NE/0132), the Health Research Authority (HRA) and the Medicines and Healthcare products Regulatory Agency (MHRA). These bodies assure that your rights, safety and wellbeing will not be compromised by participating in the ASCEnD study.

Cumbria, Northumberland, Tyne and Wear NHS Foundation Trust has reviewed all of the study documentation and has assessed the risks of this study as part of their responsibility as study Sponsor.

We have also asked people with lived experience of bipolar to review the study and study documents. This is to ensure that the study is relevant and protects your interests and rights. These individuals will continue to work with us to monitor the progress and safety of the study.

If you have a problem or if something goes wrong
If you have a concern about any aspect of this study, you can speak to a member of your study team (this could be at your hospital or clinic, or one of the research assistants) who will do their best to answer your questions.

If you are still unhappy and wish to raise your concerns with someone who is not directly involved in your care, and you are based in England or Wales, you can contact your local Patient Advice and Liaison Service (PALS).

<site to localise with local details such as PALS phone number and email address>(to remove for online generic version)

In the unlikely event that you are harmed during the study, and this is due to someone's negligence (i.e., they were careless), you may have grounds for legal action and compensation. However, you may need to pay your own legal costs. NHS Indemnity does not offer no-fault compensation (for harm that is not anyone's fault).

Your data and confidentiality
All of the information collected about and provided by you will be kept confidential.

- The study research assistants will keep a record of your contact details including name, address, email address, and telephone number, as well as your date of birth and if you are woman who may get pregnant (to ask if you might be pregnant).
- Your email address and telephone number will be used to contact you and send reminders to complete the study questionnaires. All of your information will be entered and stored on computers that are secure and password protected.

- Very occasionally, information might be given during the study that we would have a legal obligation to pass on to others (for instance, information which suggested you or others were at risk of harm). In these cases, confidentiality would be broken so that we could pass on this information to the relevant people. You would be informed of this. The study research assistants (and the Keele University researchers for those selected to take part in optional interviews only) will keep a record of emergency contact details including those for your local study team, clinical care team, GP and your next of kin, so that they can contact the relevant people if this is necessary.
- You will be given a unique study identification (ID) number instead of writing your name on study documents. Only your local study team, the study research assistants and the Keele University researchers (for those selected for optional interviews) will be able to link this number back to you using your date of birth, name and/or NHS number.
- We will ask your permission for a copy of your completed consent form to be sent securely to the Newcastle Clinical Trials Unit, to check it has been completed correctly. This is optional and you can write on your consent form if you agree to this or not.
- You will not be named in any study results, transcripts or reports on our website.
- A registry called Research+Me, which is owned by the Newcastle upon Tyne Hospitals NHS Foundation Trust, is being used to identify people who may be eligible to participate in this study. If you became involved in ASCEnD in this way, some anonymised information about you may be shared with the Newcastle upon Tyne Hospitals NHS Foundation Trust and the Research+Me team, to understand how you found out about the ASCEnD study.
- At the end of the study, all information will be kept in a secure storage area (this is called archiving) for at least 5 years. This information may be viewed throughout this time by staff working for one or more of the regulatory authorities that oversee the ASCEnD study. This makes sure any queries about the running of the study have been answered. After 5 years, all of the information will be safely destroyed.
- Fully anonymised data may be made available to other researchers both within and outside the UK to help inform other research studies. Your identity will always be protected.
- Your information will be viewed only by those who are required to do so. Your information may be looked at by authorised persons from the MHRA, Cumbria, Northumberland, Tyne and Wear NHS Foundation Trust (Study Sponsor) and Newcastle University to check that the study is being conducted to the highest standard and within legal guidelines.

If you agree and are selected to take part in an optional interview:

- Your name, address, email address and telephone number will be shared with the researchers as Keele University so that they can contact you, as well as your date of birth. Your personal details, as well as any recordings of your voice, and transcript, will be kept securely at Keele University. This is in addition to the emergency contact details described above which will only be used if there is any cause for concern during your interview. A GP who is part of the Keele University team may be in contact if there are any concerns during your interview.

- Your study ID number will be written instead of your name or any other personal details on your transcript. Only a member of the research team will be able to link your ID number back to you.
- All interview recordings will be deleted six months after the end of the optional study.
- Transcriptions may be done by the research team based at Keele University, or by a transcription company which is based in the UK but uses a USA based Sub-Processor company (SendThisFile) to assist with the transcribing process and is routinely used by researchers at Keele University.
- Your anonymised transcript will be kept on secure computers at Keele University for 5 years after the study finishes and may be used for future research if you consent to this.

If you agree to take part in the optional REWARD study:

- Your study ID and month/year of birth will used instead of your name on the Gorilla website and Newcastle University One Drive.

Your rights with regards to your data

We need to manage your information in specific ways to ensure the results from the ASCEnD study are accurate and reliable. You are free to stop taking part in the ASCEnD study at any time, without giving a reason (please see page 9 “What happens if I decide I don’t want to continue with the study?”). If you stop taking part, we will keep and use the information we have already collected about you. If you take part in the optional interview, you will have the option to request your interview is deleted until the point that it is transcribed.

You can find out more about how we use your information by:

- visiting www.hra.nhs.uk/information-about-patients/ and www.hra.nhs.uk/patientdataandresearch
- asking a member of your study team
- sending an email to the Sponsor Data Protection Officer at DPO@cntw.nhs.uk.

Results at the end of the study

At the end of the study, the results will be published in medical journals and presented in meetings to other doctors, nurses, researchers and patients. A report will be written for the study funder and put on their website. Individual results will not be fed back to you. However, you will receive a summary of the results.

It will not be possible to identify you from any study data that is published.

Where can I find out further information?

If you would like to find out more about the ASCEnD study, or have any questions, please contact your local study staff. These can be found at www.ascendtrial.co.uk/sites (for online version only) OR These are:

[Please insert contact details of the local Principal Investigator and research nurse] (for local site only)

They are also the people you should contact in the event of an emergency, if your study participation is in any way involved.

You may also find the ASCEnD website helpful: www.ascendtrial.co.uk

Thank you for reading this information sheet.
