# Supplementary material for: Open-label randomised controlled trial of aripiprazole/sertraline combination in comparison with quetiapine for the clinical and cost-effectiveness of treatment of bipolar depression (the ASCEnD study): study protocol
Source: BMJ Open. 2026 Mar 19;16(3):e112677. doi: 10.1136/bmjopen-2025-112677 (PMC13007169; doi:10.1136/bmjopen-2025-112677)
Supplement: online supplemental appendix 6 [file bmjopen-16-3-s007.pdf]

ASCEnD

Aripiprazole Sertraline Combination Effectiveness

Aripiprazole/**S**ertraline **c**ombination: clinical and cost-**e**ffectiveness in comparison with Quetiapine for the treatment of bipolar **d**epression. An open label randomised controlled trial.

The ASCEnD Study

Information Leaflet for Informal Carers (*Spouses, Family, Friends)*

INVITATION

We would like to invite you to be part of the ASCEnD Study. You have been named by someone who is taking part in the ASCEnD study as a person who supports and helps them without payment. This person has agreed that we can contact you. In this sheet we refer to anyone who provides unpaid care to someone as a 'informal carer'. We will refer to the person that you care for as the 'primary participant' in this document.

What is the ASCEnD Study?

Bipolar disorder (referred to as 'bipolar' in this document) occurs in about 1.4% of people, over a lifetime. There are currently limited treatment options available for people experiencing depressive symptoms. In addition, some of these drugs have side effects such as sedation (feeling very sleepy) and gaining weight, and often cannot be prescribed by a GP.

Although antidepressants can be very effective in people with depression and can also be prescribed by a GP, very little is known about using antidepressants in patients living with bipolar. In this study, we will use a combination of an antidepressant (sertraline) and an antipsychotic drug (aripiprazole) and compare this with an antipsychotic drug called quetiapine. Quetiapine is a common treatment for people experiencing depression in bipolar. All three drugs are already used in the NHS, but we want to find out if using a sertraline/aripiprazole combination will be beneficial in reducing depressive symptoms in people with bipolar.

Why are we interested in the experience of carers?

It has been widely acknowledged that people who are informal carers often put the needs of the person they are caring for ahead of their own. This may impact carers' health, well-being, finances, personal time and ability to work, study or undertake social activities significantly.

In 2015, approximately 8% of households in the UK had someone who was an informal carer. In 2014, it was estimated that unpaid adult carers provided care worth nearly £57 billion. Nevertheless, these costs and impacts on the well-being of people who provide informal care are not regularly measured or assessed.

As part of the ASCEnD study, we would like to better understand the experience and impact of being an informal carer for someone with bipolar disorder. We hope that these results will help to address the needs of informal carers better in the future and contribute to a deeper understanding of the potential benefits and value of the investigated treatment options.

Why have I been invited to take part in ASCEnD?

You have been identified as a person who provides informal care by a participant who has consented to take part in the ASCEnD study. They have given their consent for you to take part in the carer part of the study. You also have the ability to complete questionnaires online.

Do I have to take part?

No. You can decide if you want to take part. If you agree to take part, you can change your mind and withdraw from the study at any time independently from the participation of the primary participant without having to give a reason. How you or the primary participant are treated will not change in anyway. The primary participant can still take part in the study even if you do not wish to take part.

What would taking part involve?

If you decide to take part, you will be:

1. Asked to sign a consent form. You will receive a copy of this.
2. Asked to provide some information (including your initials, age, sex, gender, marital status, education level, ethnicity and post-code).
3. Asked to complete four questionnaires to help us better understand the well-being impacts and costs of caring for a person with bipolar disorder.
4. Registered with an online system called ePRO, where you will complete the same questionnaires at later time points.

Completing questionnaires
We will ask you to complete questionnaires online via ePRO at 4 weeks, 14 weeks and 24 weeks after the primary participant's screening/baseline appointment. You will receive reminders via email and text message to complete your questionnaires.

Each time you access questionnaires on ePRO you will be asked for a memorable word that you provided during registration. **It is important that you do not share your memorable word with anyone**.

What are the possible benefits and risks of taking part?

Your help will greatly benefit our understanding of the impact and experience of caring for a person with bipolar.

We think there are minimal risks to being part of the study as you will be asked to complete questionnaires at 4 different timepoints and provide some information about yourself. If you find completing the questionnaires difficult or upsetting, please contact the primary participant's study team using the contact details at the end of this leaflet.

Is there anything else I need to know?

Organisation and funding of the study

<u>Chief Investigator:</u> The doctor in charge of the study is Dr Stuart Watson, a Consultant Psychiatrist. He works for Newcastle University and at Cumbria, Northumberland, Tyne and Wear NHS Foundation Trust.

<u>Study Sponsor:</u> Cumbria, Northumberland, Tyne and Wear NHS Foundation Trust is the sponsor of the study. This means they have overall responsibility. The study is managed by the Newcastle Clinical Trials Unit, a part of Newcastle University, on behalf of the Sponsor. Researchers from Keele University are organising and conducting the optional interviews.

<u>Study Funders:</u> The study has received funding as part of the National Institute for Health Research (NIHR) Health Technology Assessment (HTA) programme (reference NIHR132773). The NIHR is funded by the UK Government to carry out research for the benefit of the NHS and its patients.

A mental health research charity called the McPin Foundation (www.mcpin.org) is supporting the involvement of people with lived experience of bipolar in this study.

Ten NHS Hospital Trusts will be taking part in this study. Each trust will have a lead study doctor, called a Principal Investigator. The Principal Investigator at your NHS Trust is ____________________________ (to remove for online generic version)

Review of the study

The study has been reviewed by independent experts from the NIHR. They ensure the study is an accurate way to understand whether an aripiprazole/sertraline combination may be beneficial to individuals experiencing bipolar depression.

The study has also been reviewed by, and received approval from, the North East-Newcastle and North Tyneside Research Ethics Committee 1 (REC Reference: 23/NE/0132) Research Ethics Committee, the Health Research Authority (HRA) and the Medicines and Healthcare products Regulatory Agency (MHRA). These bodies assure that your rights, safety and wellbeing will not be compromised by participating in the ASCEnD study.

Cumbria, Northumberland, Tyne and Wear NHS Foundation Trust has reviewed all of the study documentation and has assessed the risks of this study as part of their responsibility as study Sponsor.

We have also asked people with lived experience of bipolar to review the study and study documents. This is to ensure that the study is relevant and protects your interests and rights. These individuals will continue to work with us to monitor the progress and safety of the study.

If you have a problem or if something goes wrong

If you have a concern about any aspect of this study, you can speak to a member of the primary participant's study team using the contact details at the end of this leaflet. These people will do their best to answer your questions.

If you are still unhappy and wish to raise your concerns with someone who is not directly involved in the care of the primary participant, and you are based in England or Wales, you can contact your local Patient Advice and Liaison Service (PALS).

<site to localise with local details such as PALS phone number and email address>____________ (to remove for online generic version)

In the unlikely event that you are harmed during the study, and this is due to someone's negligence (i.e., they were careless), you may have grounds for legal action and compensation. However, you may need to pay your own legal costs. NHS Indemnity does not offer no-fault compensation (for harm that is not anyone's fault).

Your data and confidentiality

All of the information collected about and provided by you will be kept confidential. We will not share your answers to the questionnaires with the primary participant or share their information with you.

- Your email address and telephone number will be used to contact you and send reminders to complete the study questionnaires. All of your information will be entered and stored on computers that are secure and password protected.
- You will be given a unique study identification (ID) number instead of writing your name on study documents. Only the staff at your hospital or clinic will be able to link this number back to you using your date of birth, name and NHS number.
- You will not be named in any study results, transcripts, reports or on our website.
- At the end of the study, all information will be kept in a secure storage area (this is called archiving) for at least 5 years. This information may be viewed throughout this time by staff working for one or more of the regulatory authorities that oversee the

ASCEnD study. This makes sure any queries about the running of the study have been answered. After 5 years, all of the information will be safely destroyed.

- Fully anonymised data may be made available to other researchers both within and outside the UK to help inform other research studies. Your identity will always be protected.
- Your information will be viewed only by those who are required to do so. Your information may be looked at by authorised persons from the MHRA, Cumbria, Northumberland, Tyne and Wear NHS Foundation Trust (Study Sponsor) and Newcastle University to check that the study is being conducted to the highest standard and within legal guidelines.

Your rights with regards to your data

We need to manage your information in specific ways to ensure the results from the ASCEnD study are accurate and reliable. You are free to stop taking part in the ASCEnD study at any time, without giving a reason. This will not affect how we treat you or the primary participant. If you stop taking part, we will keep and use the information we have already collected about you. You do not need to explain your reasons, but it is helpful to the study if you do.

You can find out more about how we use your information by:

- Visiting www.hra.nhs.uk/information-about-patients/ and www.hra.nhs.uk/patientdataandresearch
- asking a member of the primary participant's study team
- sending an email to the Sponsor Data Protection Officer at DPO@cntw.nhs.uk

Results at the end of the study

At the end of the study, the results will be published in medical journals and presented in meetings to other doctors, nurses, researchers and patients. A report will be written for the study funder and put on their website. Individual results will not be fed back to you. However, you will receive a summary of the results.

It will not be possible to identify you from any study data that is published.

Where can I find out further information?

If you would like to find out more about the ASCEnD study, or have any questions, please contact the primary participant's local study staff. These can be found at www.ascendtrial.co.uk/sites (for online version only) OR These are:

[Please insert contact details of the local Principal Investigator and research nurse]

You may also find the ASCEnD website helpful: www.ascendtrial.co.uk

Thank you for reading this information sheet.
