# Supplementary material for: Open-label randomised controlled trial of aripiprazole/sertraline combination in comparison with quetiapine for the clinical and cost-effectiveness of treatment of bipolar depression (the ASCEnD study): study protocol
Source: BMJ Open. 2026 Mar 19;16(3):e112677. doi: 10.1136/bmjopen-2025-112677 (PMC13007169; doi:10.1136/bmjopen-2025-112677)
Supplement: online supplemental appendix 7 [file bmjopen-16-3-s008.pdf]

Medication Guide for Clinicians

Introduction

For the purposes of this trial, quetiapine, sertraline and aripiprazole are classed as investigational medicinal products (IMP). Prescribing should follow standard clinical practice. Quetiapine is being used within its licensed indication in this study. Sertraline and aripiprazole are being used outside of their licensed indications. Participants will be randomised to open-label aripiprazole/sertraline combination or quetiapine. Participants who discontinue their randomised trial medication will be encouraged to remain in the trial and to continue to provide outcome data.

Screening and Consent

A key eligibility criterion is that there is clinical uncertainty regarding the next course of treatment and judgement that the aripiprazole/sertraline combination and quetiapine treatment arms are both clinically appropriate and represent equipoise.

Randomisation

The researcher may or may not be the prescribing clinician and the prescribing clinician may or may not be in a position to start the next course of treatment immediately. It may be that drug rationalisation is required before IMP can start. If eligible, the patient can, and should, be randomised regardless of whether or not the randomised medication can start immediately. We are interested in what happens to the patient after the decision to commence aripiprazole/sertraline or quetiapine is made.

Patients on an antidepressant at randomisation

You may consider the study for patients who are already prescribed an antidepressant. If the patient meets eligibility criteria (e.g., has bipolar depression with a QIDS-SR>10 and there is clinical equipoise), then the patient can be randomised. If randomised to quetiapine, then a clinical decision about whether or not to continue the antidepressant can be made. If randomised to aripiprazole/sertraline then standard practice will be to switch any current antidepressant to sertraline (though if it makes pragmatic clinical sense, other current antidepressants that are compatible with sertraline and aripiprazole, e.g., mirtazapine, may be continued and combined with the sertraline).

Patients on an antipsychotic at randomisation

It is possible that a patient may be taking quetiapine and still meet eligibility criteria. This may occur for instance if there appears to have been a partial response to quetiapine and you and your patient are in two minds whether to continue this drug (because of its partial value) or to switch to seek a better response. The crucial question remains whether quetiapine and aripiprazole/sertraline combination represents clinical equipoise. In this instance, if randomised to quetiapine then the dose should be altered upwards or downwards (see dosage section) if clinically appropriate to 300mg by making stepwise changes. Similarly, a patient may be on aripiprazole at baseline and similar logic should be followed (see dosage section). Patients may also be prescribed a different antipsychotic at randomisation. It is anticipated that this will usually be switched (as per standard clinical practice) to aripiprazole or quetiapine in the post-randomisation period.

Prescribing

IMP will be provided, open label, by local pharmacies according to their usual prescribing practices. Any brand of study medication may be used.

The study does not mandate use of specific medication dosages or dose escalation procedures. Instead, clinicians are encouraged to use their judgement in accordance with clinical guidelines, the BNF dose schedule and this guidance. Dosage decisions and decisions regarding the ongoing use of study medications taken during the study remain the responsibility of the prescribing clinician. The following guidance should be followed where possible, but clinicians may choose a dosage escalation protocol more suited to the participant's clinical needs.

Aripiprazole/Sertraline dosage

The BNF recommends a dose range for sertraline of 50-200 mg daily for individuals experiencing depression and 15-30mg aripiprazole daily for individuals with bipolar disorder. However, for aripiprazole, research evidence suggests effectiveness of lower doses, up to 7.5mg daily, for antidepressant augmentation in MDD(1-5), in MDD with mixed features(6) and in bipolar depression(7). The side-effect profile of these drugs is largely dose dependent. For sertraline, a recent dose response meta-analysis suggests that 100mg offers greater benefit than 50mg(8), whereas studies that combine sertraline with the other SSRIs suggest either that there is (9, 10) or isn't(11) a dose response efficacy benefit above 50mg sertraline. The evidence of efficacy of aripiprazole monotherapy or sertraline monotherapy in bipolar depression is not clear. It is the combination that is of interest in this trial.

Therefore, starting daily dosages of 50 mg sertraline and 2.5 mg aripiprazole are recommended for participants in ASCEnD. It is recommended that, usually, both drugs at these doses are started together. However, it is entirely within the ethos of the trial if you and your patient would prefer to start the drugs separately. Similarly, 25mg rather than 50mg is an acceptable sertraline starting dose. Co-prescription of diazepam and zopiclone may be considered during initiation and dose escalation, in keeping with safe practice and BNF limits (usually for maximum of 2-week period).

For sertraline, it is anticipated that 100mg will be more likely to be effective than 50mg and so we recommend that, after a week, if the drugs are tolerated and there isn't a trajectory of improvement, that sertraline be doubled from 50 mg to 100mg (or from 25 to 50mg). This can, perhaps, most pragmatically be done by prescribing 50 mg for one week and 100mg for subsequent weeks and asking for this to be dispensed in 50mg tablets to allow flexibility (with the decision to step-up to 100mg being made by patient and/or prescriber). Thereafter, a lack of clinical response (if tolerability allows), should prompt up-titration in fortnightly steps of 50 mg sertraline or 1.25 or 2.5mg aripiprazole (alternating which drug is increased at each step) to a maximum of 150 mg and 7.5 mg daily respectively. The preferred aripiprazole step is 1.25 rather than 2.5 mg. Sertraline 200mg and/or aripiprazole 10mg would be reasonable if there is a lack of response to lower doses but please note that, particularly for aripiprazole, the higher dose is more likely to cause side effects and seems unlikely to offer additional therapeutic benefit.

Please be aware that the smallest currently available aripiprazole tablet is 5mg. Participants (and pharmacists) should be advised that to obtain a 2.5mg dose, a scored tablet may be halved each morning or, because of aripiprazole's long half-life, a 5mg tablet may be taken on alternating days. Similarly, 3.75mg can be attained as 5mg for two days in three or alternating between 2.5mg and 5mg.

Quetiapine dosage

The BNF recommends a dose range for quetiapine of 300-600 mg daily for individuals experiencing bipolar depression. Quetiapine daily doses in clinical trials typically start at 50 mg and increase to 300 mg. ASCEnD will rely on guidance from the CEQUEL study which achieved a dose of 300 mg by day 7 (50 mg on days 1 and 2, 100mg on days 3 and 4, 200mg on days 5 and 6, and then 300mg)(12). Bigger doses (i.e., between 300 and 600mg) may be used, particularly in response to manic symptoms, though doses closer to 300mg may have the right balance between efficacy and tolerability. This regime is similar but not identical to the LQD quetiapine regime(13). It is quicker than often done in clinical practice, but the evidence suggests that this speed of up titration is well tolerated. Quetiapine is available in an immediate release (IR) and in an extended release (XL) formulation. The choice between IR and XL will remain a clinical decision in participants randomised to quetiapine.

Mania prophylaxis and treatment

1 mg aripiprazole daily produces 50% occupancy of D2 receptors at steady state (which is reached after 2–3 weeks of regular doses); 10 mg daily produces 95% occupancy (14) . It has been estimated that people with mild manic states require 50–60% of D_2 and/or D_3 receptor blockade, whereas 95% receptor blockade is required for severe mania(15). The partial agonist aripiprazole has 25% intrinsic activity (i.e., full dose aripiprazole induces a quarter of the agonism of a full agonist (at full dose) and 75% of the antagonism of a full antagonist) (14) suggesting that 10mg aripiprazole should be adequate to treat hypomania but high dose aripiprazole will not be able to attain the dopamine receptor blockade required to treat severe mania. Whilst meta-analysis shows that aripiprazole reduces the risk of manic relapse(16), this evidence is based on aripiprazole depot(17) and studies using aripiprazole doses of at least 10mg. The evidence base doesn't convincingly therefore tell us whether the low dose aripiprazole we are recommending in this study will offer prophylaxis against manic relapse. Quetiapine at 300mg has been shown to reduce relapse to mania risk. Higher doses may be needed to treat mania.

We would encourage you to help all patients in the trial recognise early signs of manic relapse, and. for patients taking aripiprazole, to use temporary increase in aripiprazole doses (10mg usually, but 15mg if needed) to treat emerging hypomania. Severe mania will most likely require an alternate/additional antimanic agent.

Adverse Effects, Cautions and Contra-indications

Local site teams should follow their usual clinical practice and consult the BNF to review side-effects, cautions and contra-indications. For example:

- Aripiprazole can cause dose dependent side-effects including GI disorders, headache, sleep disturbance, drowsiness, and akathisia. Pathological gambling has been sometimes reported.
- Sertraline can reduce libido and cause impotence and anorgasmia. It can be associated with GI disturbance, restlessness, insomnia, drowsiness, sweating, hyponatraemia and GI bleeds (particularly when taken with NSAIDs).
- Quetiapine use can be associated with somnolence, headache and dizziness, weight gain, hyperglycaemia, rises in plasma triglyceride and total cholesterol, increased appetite, raised liver enzymes, leucopaenia, neutropaenia and pancytopaenia.

Aripiprazole, sertraline and quetiapine are metabolised in the liver and so caution is needed in severe hepatic impairment. Aripiprazole, sertraline and quetiapine metabolism relies on the P450 isoenzymes including CYP3A4 and CYP2D6 (for aripiprazole, the CYP2D6 status is a major determinant of metabolism) therefore dose adjustment is recommended when concomitant inducers or inhibitors are used. Many of the antidepressants (bupropion, citalopram, (nor)fluoxetine, duloxetine, escitalopram, paroxetine, sertraline, moclobemide) are CYP3A4 and CYP2D6 inhibitors.

Regarding cardiac risk, tachycardia, orthostatic hypotension, bradycardia, ventricular arrhythmia, cardiac arrest and sudden unexpected death have been only very rarely recorded with aripiprazole. Orthostatic hypotension has been reported with sertraline. QT-interval prolongation is uncommon with quetiapine at therapeutic doses. For all three, however, it is sensible to be cautious when co-prescribing drugs which may prolong the QTc interval or cause electrolyte imbalance.

Seizures have been rarely reported with aripiprazole, quetiapine and sertraline.

It is better to avoid abrupt withdrawal of all ASCEnD drugs. If a drug needs to be discontinued then please manage your patient as usual- e.g., if sertraline causes side effects, a different antidepressant may be in order.

NICE 2021 Recommendation for monitoring of someone taking antipsychotics

Baseline measures should be taken in secondary care. The BNF doesn't list these but does state that regular monitoring may be done in primary care and may include:

Body weight, or body mass index (BMI) - weekly for the first 6 weeks, then at 3 months. Thereafter every 12 months, or more often if the person is gaining weight rapidly.

Serum electrolytes and urea including creatinine and estimated glomerular filtration rate every 12 months.

Full blood count every 12 months.

Blood lipids, 3 months after starting treatment, then every 12 months.

Plasma glucose or HbA1c, at 3 months after starting treatment, then every 12 months. Ask about symptoms of hyperglycaemia (such as polydipsia, polyuria, and increased appetite).

Pulse and blood pressure, during dose titration and at each dose change for quetiapine but not required for aripiprazole.

ECG

The NICE 2014 psychosis guidance states that Before starting antipsychotic medication, offer the person with psychosis or schizophrenia an electrocardiogram (ECG) if specified in the summary of product characteristics (it isn't for either aripiprazole or quetiapine). a physical examination has identified specific cardiovascular risk (such as diagnosis of high blood pressure), there is a personal history of cardiovascular disease or the service user is being admitted as an inpatient. [2009]. The NICE 2021 monitoring of antipsychotics page cites the 2014 guidance but doesn't discuss baseline use. For monitoring it states that electrocardiography (ECG) should be used after dose changes and, ideally, also annually, but that this is not required for antipsychotics with no effect, or a low-to-moderate effect on the QT interval (The Maudsley Prescribing Guidelines categorise aripiprazole as low effect and

quetiapine as moderate effect on QTc) and where there are no other risk factors for arrhythmia. The aripiprazole and quetiapine SPCs don't specify baseline ECG. Aripiprazole | Drugs | BNF | NICE. The BNF pages for aripiprazole and quetiapine both state, in the caution section, "An ECG may be required, particularly if physical examination identifies cardiovascular risk factors, personal history of cardiovascular disease, or if the patient is being admitted as an inpatient". Local guidance may differ.

Further information

If discussion would be helpful about this guidance in general or for a particular (potential) participant, please contact the ASCEnD team via ASCEnD@newcastle.ac.uk and/or the Chief Investigator via stuart.watson@newcastle.ac.uk. If your query concerns an individual, please do not include personal identifiable information in your email. Out of hours, please contact CNTW Initial Response Service 0303 123 1145.

If your experience suggests that this guidance should evolve (e.g., if you have a clinical dilemma not covered by the guidance) then please let us know.

Refs

1. Kamijima K, Higuchi T, Ishigooka J, Ohmori T, Ozaki N, Kanba S, et al. Aripiprazole augmentation to antidepressant therapy in Japanese patients with major depressive disorder: A randomized, double-blind, placebo-controlled study (ADMIRE study). J Affect Disord. 2013;151(3):899-905.
2. Furukawa Y, Hamza T, Cipriani A, Furukawa TA, Salanti G, Ostinelli EG. Optimal dose of aripiprazole for augmentation therapy of antidepressant-refractory depression: Preliminary findings based on a systematic review and dose–effect meta-analysis. The British Journal of Psychiatry. 2022;221(2):440-7.
3. Habermann TJ, Singh B. Long-term Efficacy and Tolerability of Adjunctive Aripiprazole for Major Depressive Disorder. 2021.
4. Lenze EJ, Mulsant BH, Roose SP, Lavretsky H, Reynolds III CF, Blumberger DM, et al. Antidepressant augmentation versus switch in treatment-resistant geriatric depression. New Engl J Med. 2023;388(12):1067-79.
5. Shin C, Pae CU, Kwak KP, Jeon SW, Jeong HG, Kim JW, et al. Additional Reduction of Residual Symptoms with Aripiprazole Augmentation in the Patients with Partially Remitted Major Depressive Disorder. Clin Psychopharmacol Neurosci. 2021;19(2):243-53.
6. Han C, Wang SM, Bahk WM, Lee SJ, Patkar AA, Masand PS, et al. The Potential Utility of Aripiprazole Augmentation for Major Depressive Disorder with Mixed Features Specifier: A Retrospective Study. Clin Psychopharmacol Neurosci. 2019;17(4):495-502.
7. Kelly T, Lieberman DZ. The Utility of Low-Dose Aripiprazole for the Treatment of Bipolar II and Bipolar NOS Depression. J Clin Psychopharmacol. 2017;37(1):99-101.
8. Luo X, Zhu D, Li J, Ren M, Liu Y, Si T, et al. Selection of the optimal dose of sertraline for depression: a dose-response meta-analysis of randomized controlled trials. Psychiatry Res. 2023:115391.
9. Furukawa TA, Cipriani A, Cowen PJ, Leucht S, Egger M, Salanti G. Optimal dose of selective serotonin reuptake inhibitors, venlafaxine, and mirtazapine in major depression: a systematic review and dose-response meta-analysis. The Lancet Psychiatry. 2019;6(7):601-9.
10. Hieronymus F, Nilsson S, Eriksson E. A mega-analysis of fixed-dose trials reveals dose-dependency and a rapid onset of action for the antidepressant effect of three selective serotonin reuptake inhibitors. Translational psychiatry. 2016;6(6):e834-e.
11. Johnson CF, Maxwell M, Williams B, Dougall N, MacGillivray S. Dose-response effects of selective serotonin reuptake inhibitor monotherapy for the treatment of depression: systematic review of reviews and meta-narrative synthesis. BMJ medicine. 2022;1(1).

12. Geddes JR, Gardiner A, Rendell J, Voysey M, Tunbridge E, Hinds C, et al. Comparative evaluation of quetiapine plus lamotrigine combination versus quetiapine monotherapy (and folic acid versus placebo) in bipolar depression (CEQUEL): a 2 x 2 factorial randomised trial. The lancet Psychiatry. 2016;3(1):31-9.
13. Marwood L, Taylor R, Goldsmith K, Romeo R, Holland R, Pickles A, et al. Study protocol for a randomised pragmatic trial comparing the clinical and cost effectiveness of lithium and quetiapine augmentation in treatment resistant depression (the LQD study). BMC psychiatry. 2017;17(1):231.
14. Cookson J, Pimm J. Partial agonists of dopamine receptors: receptor theory and the dopamine hypothesis of psychosis. BJPsych Advances. 2023;29(2):141-4.
15. Chou JC-Y, Czobor P, Charles O, Tuma I, Winsberg B, Allen MH, et al. Acute Mania: Haloperidol Dose and Augmentation With Lithium or Lorazepam. J Clin Psychopharmacol. 1999;19(6):500-5.
16. Kishi T, Ikuta T, Matsuda Y, Sakuma K, Okuya M, Mishima K, et al. Mood stabilizers and/or antipsychotics for bipolar disorder in the maintenance phase: a systematic review and network meta-analysis of randomized controlled trials. Mol Psychiatry. 2021;26(8):4146-57.
17. Calabrese JR, Sanchez R, Jin N, Amatniek J, Cox K, Johnson B, et al. Efficacy and safety of aripiprazole once-monthly in the maintenance treatment of bipolar I disorder: a double-blind, placebo-controlled, 52-week randomized withdrawal study. The Journal of clinical psychiatry. 2017;78(3):1349.
