# Supplementary material for: Open-label randomised controlled trial of aripiprazole/sertraline combination in comparison with quetiapine for the clinical and cost-effectiveness of treatment of bipolar depression (the ASCEnD study): study protocol
Source: BMJ Open. 2026 Mar 19;16(3):e112677. doi: 10.1136/bmjopen-2025-112677 (PMC13007169; doi:10.1136/bmjopen-2025-112677)
Supplement: online supplemental appendix 8 [file bmjopen-16-3-s009.pdf]

To be printed on local headed paper

ASCEnD
Aripiprazole Sertraline Combination Effectiveness

Guide to Red Pill self-service (ePRO) questionnaire completion

Version: 2.0

Date: 26th September 2024

Author: Chris Weetman (Data Manager)

1 Instructions on self-service questionnaire completion

All screenshot examples shown have been taken from the ASCEnD Red Pill test database and contain additional text that highlights the use of the test system that would not be present when using the live database.

1.1 Welcome Message

The following welcome message is sent out automatically by the Red Pill system once a participant/carer has been invited to complete forms via the self-service function:

[ASCEnD - TEST] Welcome to the ASCEnD trial

From: **automated@sealedenvelope.com**, To: **uunphucg**, Date **2024-06-05 13:42:04**

Thank you for agreeing to take part in the ASCEnD trial. We will contact you again when we have questionnaires we would like you to complete.

Many thanks
The trial team
Aripiprazole/sertraline combination: clinical and cost-effectiveness in comparison with quetiapine for the treatment of bipolar depression.

** WARNING! This message was sent during testing and should not be acted upon **

ASCEnD
Aripiprazole/sertraline combination: clinical and cost-effectiveness in comparison with quetiapine for the treatment of bipolar depression.

Note, this message was auto-generated on 5 Jun 2024 13:41 (UTC) and was sent from an email address that does not accept replies.

Sealed Envelope
www.sealedenvelope.com

Here is an example of an informal carer welcome screen:

[ASCEnD - TEST] Welcome to the ASCEnD trial

From: **automated@sealedenvelope.com**, To: **vbmhtkpx**, Date **2024-06-26 13:17:08**

Thank you for agreeing to take part in the ASCEnD trial. We will contact you again when we have questionnaires we would like you to complete.

Many thanks
The trial team
Aripiprazole/sertraline combination: clinical and cost-effectiveness in comparison with quetiapine for the treatment of bipolar depression.

** WARNING! This message was sent during testing and should not be acted upon **

ASCEnD
Aripiprazole/sertraline combination: clinical and cost-effectiveness in comparison with quetiapine for the treatment of bipolar depression.

Note, this message was auto-generated on 26 Jun 2024 13:16 (UTC) and was sent from an email address that does not accept replies.

Sealed Envelope
www.sealedenvelope.com

1.2 Invitation, selection and completion of online forms

Upon randomisation of a participant an invitation will be sent that contains a unique link to complete follow-up week 1 questionnaires. Informal carers will receive an invitation 28 days after the linked participant's randomisation took place; carers must be marked as consented and eligible in order to answer the questionnaires.

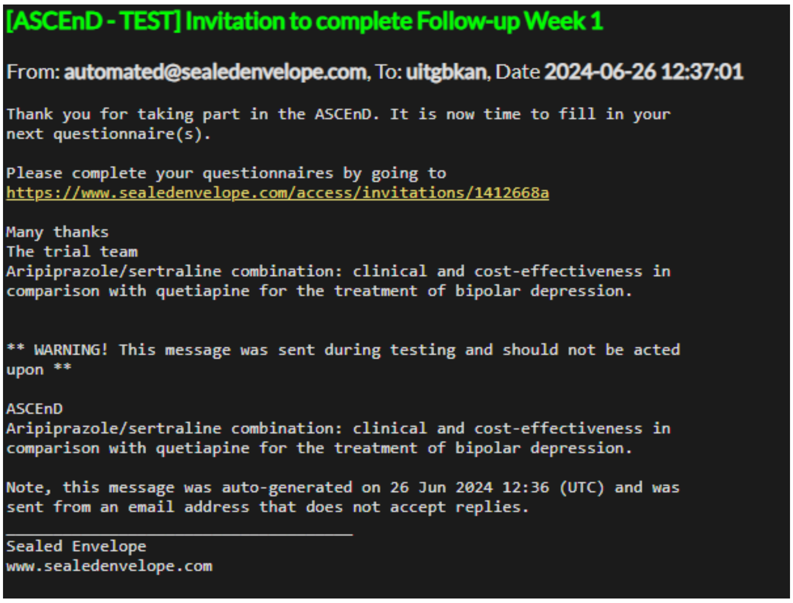

[ASCEnD - TEST] Invitation to complete Follow-up Week 1

From: **automated@sealedenvelope.com**, To: **uitgbkan**, Date **2024-06-26 12:37:01**

Thank you for taking part in the ASCEnD. It is now time to fill in your next questionnaire(s).

Please complete your questionnaires by going to https://www.sealedenvelope.com/access/invitations/1412668a

Many thanks
The trial team
Aripiprazole/sertraline combination: clinical and cost-effectiveness in comparison with quetiapine for the treatment of bipolar depression.

** WARNING! This message was sent during testing and should not be acted upon **

ASCEnD
Aripiprazole/sertraline combination: clinical and cost-effectiveness in comparison with quetiapine for the treatment of bipolar depression.

Note, this message was auto-generated on 26 Jun 2024 12:36 (UTC) and was sent from an email address that does not accept replies.

Sealed Envelope
www.sealedenvelope.com

Upon clicking the unique link, the welcome screen will appear. Here is an example of a participant welcome screen:

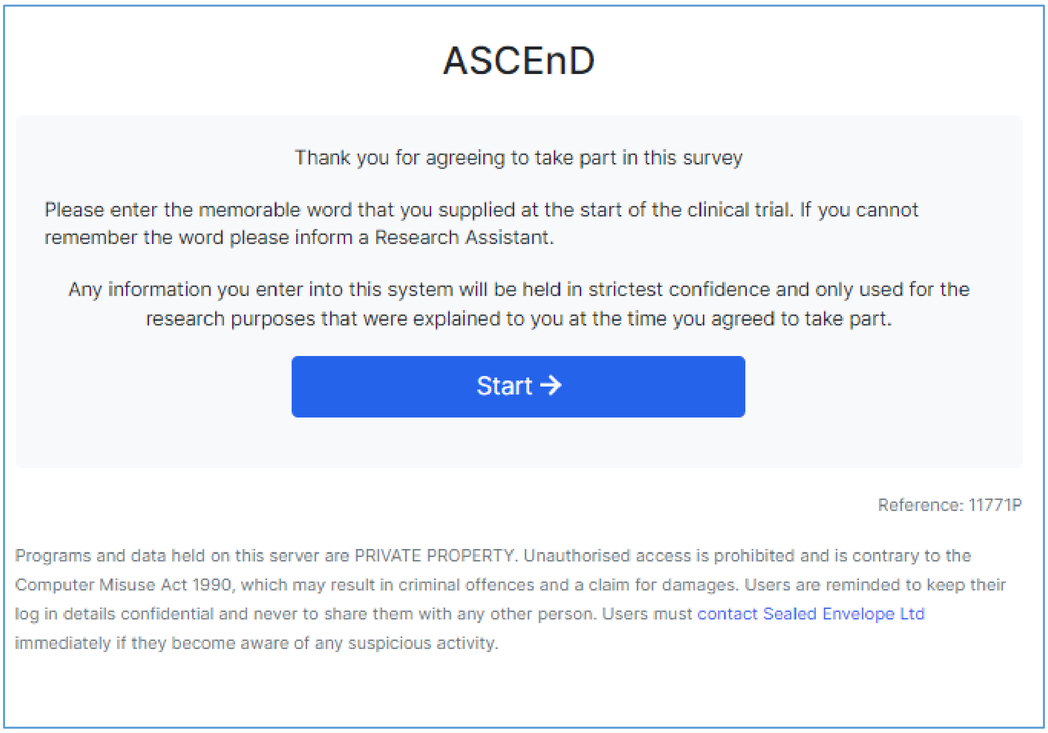

ASCEnD

Thank you for agreeing to take part in this survey

Please enter the memorable word that you supplied at the start of the clinical trial. If you cannot remember the word please inform a Research Assistant.

Any information you enter into this system will be held in strictest confidence and only used for the research purposes that were explained to you at the time you agreed to take part.

Start →

Reference: 11771P

Programs and data held on this server are PRIVATE PROPERTY. Unauthorised access is prohibited and is contrary to the Computer Misuse Act 1990, which may result in criminal offences and a claim for damages. Users are reminded to keep their log in details confidential and never to share them with any other person. Users must contact Sealed Envelope Ltd immediately if they become aware of any suspicious activity.

Newcastle University

To be printed on local headed paper

After the ‘start’ button is clicked, the questionnaire selection screen will load. Here is an example of a participant questionnaire selection screen (the number of available questionnaires will vary depending upon the number of weeks since randomisation):

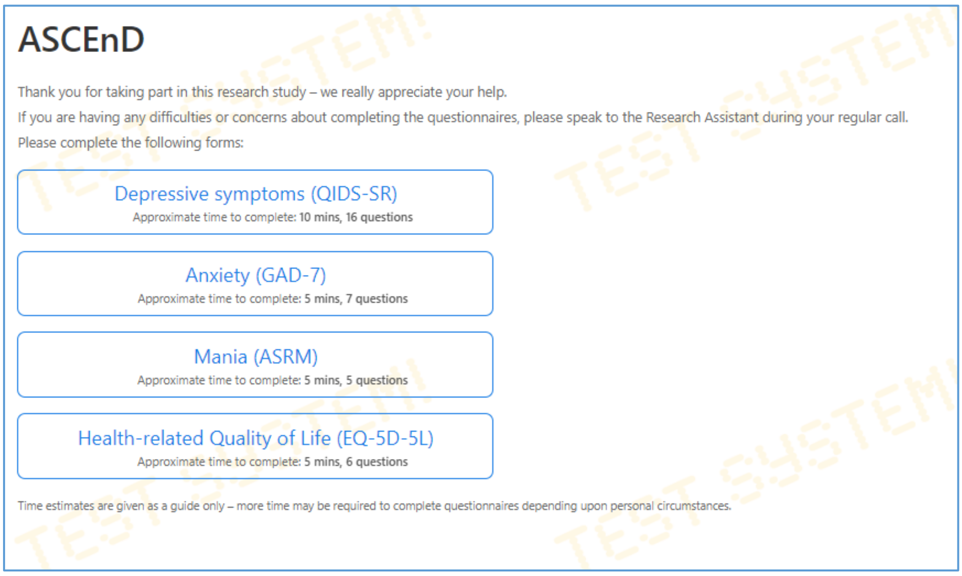

Clicking on a questionnaire title will allow responses to be entered for that questionnaire, some questionnaires may be several pages long and some may only be a single page. The number of questions and estimated completion time is provided for each questionnaire on the selection page, but the time taken will vary from person to person and depending upon the answers given. Here is an example of a questionnaire response page:

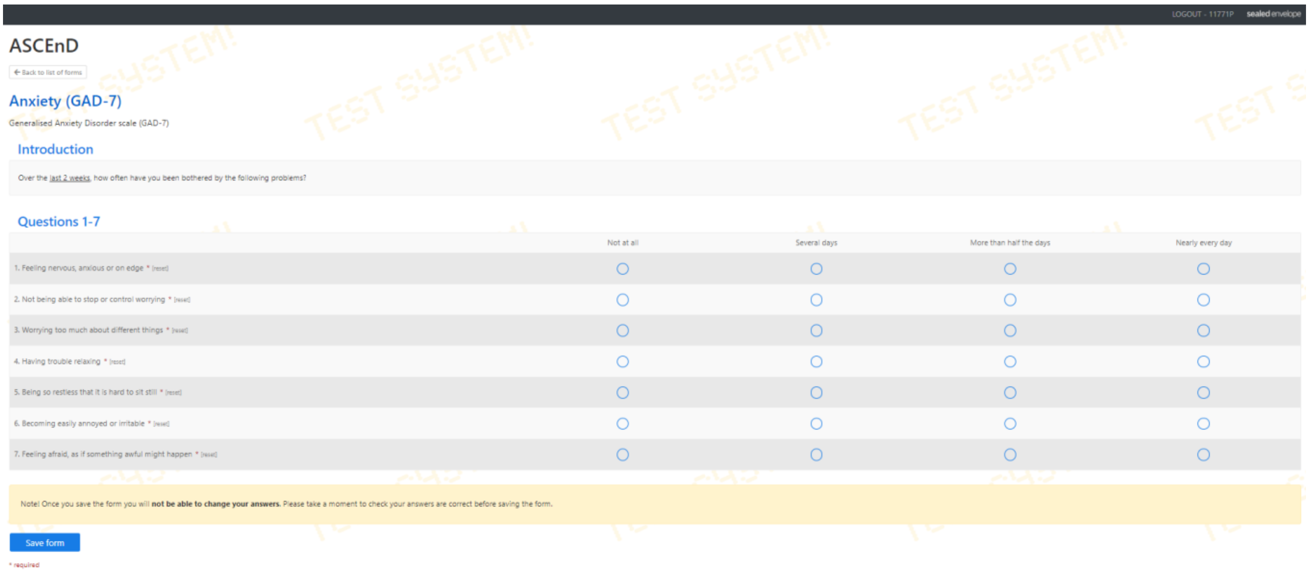

1.3 Logging out and saving data

The **LOGOUT** link will allow a user to securely leave the page and log back in at a later date by re-using the unique link included in the email/text message. **Each link is only valid for 7 days after it was first sent** – after 7 days the link will expire and a different link will be sent for the next 7 days of the trial.

The **Save form** button will appear at the bottom of the final page of each questionnaire. **If the save form button has not been pushed, responses will be lost if the logout button is pushed, or the browser window is closed without saving.**
