# Supplementary material for: Open-label randomised controlled trial of aripiprazole/sertraline combination in comparison with quetiapine for the clinical and cost-effectiveness of treatment of bipolar depression (the ASCEnD study): study protocol
Source: BMJ Open. 2026 Mar 19;16(3):e112677. doi: 10.1136/bmjopen-2025-112677 (PMC13007169; doi:10.1136/bmjopen-2025-112677)
Supplement: online supplemental appendix 9 [file bmjopen-16-3-s010.pdf]

Participant ID: ___ ___ ___ ___ ___ P

Date of visit (DD/MM/YYYY): __ __ / __ __ / __ __ __ __

ASCEnD

Aripiprazole Sertraline Combination Effectiveness

Patient Questionnaire Booklet

For use at the baseline visit

Anxiety (GAD-7)

GAD-7

Over the <u>last 2 weeks</u>, how often have you been bothered by the following problems? *(Use "✔" to indicate your answer)*	Not at all	Several days	More than half the days	Nearly every day
**1.** Feeling nervous, anxious or on edge	0	1	2	3
**2.** Not being able to stop or control worrying	0	1	2	3
**3.** Worrying too much about different things	0	1	2	3
**4.** Having trouble relaxing	0	1	2	3
**5.** Being so restless that it is hard to sit still	0	1	2	3
**6.** Becoming easily annoyed or irritable	0	1	2	3
**7.** Feeling afraid, as if something awful might happen	0	1	2	3

Mania (ASRM)

1. **Please read each group of statements/question carefully.**
2. Choose the one statement in each group that best describes the way you (the individual receiving care) have been feeling for **the past week**.
3. Check the box (✓ or x) next to the statement selected.
4. **Please note:** The word "occasionally" when used here means once or twice; "often" means several times or more and "frequently" means most of the time.

**Question 1**	
	I do not feel happier or more cheerful than usual;
	I occasionally feel happier or more cheerful than usual;
	I often feel happier or more cheerful than usual;
	I feel happier or more cheerful than usual most of the time;
	I feel happier or more cheerful than usual all of the time;
**Question 2**	
	I do not feel more self-confident than usual;
	I occasionally feel more self-confident than usual;
	I often feel more self-confident than usual;
	I frequently feel more self-confident than usual;
	I feel extremely self-confident all of the time;
**Question 3**	
	I do not need less sleep than usual;
	I occasionally need less sleep than usual;
	I often need less sleep than usual;
	I frequently need less sleep than usual;
	I can go all day and all night without any sleep and still not feel tired;
**Question 4**	
	I do not talk more than usual;
	I occasionally talk more than usual;
	I often talk more than usual;
	I frequently talk more than usual;
	I talk constantly and cannot be interrupted;
**Question 5**	
	I have not been more active (either socially, sexually, at work, home, or school) than usual;
	I have occasionally been more active than usual;
	I have often been more active than usual;
	I have frequently been more active than usual;
	I am constantly more active or on the go all the time;

Copyright © 1997, with permission from Elsevier.

Participant ID: ___ ___ ___ ___ ___ P

Date of visit (DD/MM/YYYY): __ __ / __ __ / __ __ __ __

EQ-5D-5L

Health Questionnaire

English version for the UK

© 2009 EuroQol Research Foundation. EQ-5D™ is a trade mark of the EuroQol Research Foundation. UK (English) v1.2

Under each heading, please tick the ONE box that best describes your health TODAY.

MOBILITY

I have no problems in walking about ❑
I have slight problems in walking about ❑
I have moderate problems in walking about ❑
I have severe problems in walking about ❑
I am unable to walk about ❑

SELF-CARE

I have no problems washing or dressing myself ❑
I have slight problems washing or dressing myself ❑
I have moderate problems washing or dressing myself ❑
I have severe problems washing or dressing myself ❑
I am unable to wash or dress myself ❑

USUAL ACTIVITIES *(e.g. work, study, housework, family or leisure activities)*

I have no problems doing my usual activities ❑
I have slight problems doing my usual activities ❑
I have moderate problems doing my usual activities ❑
I have severe problems doing my usual activities ❑
I am unable to do my usual activities ❑

PAIN / DISCOMFORT

I have no pain or discomfort ❑
I have slight pain or discomfort ❑
I have moderate pain or discomfort ❑
I have severe pain or discomfort ❑
I have extreme pain or discomfort ❑

ANXIETY / DEPRESSION

I am not anxious or depressed ❑
I am slightly anxious or depressed ❑
I am moderately anxious or depressed ❑
I am severely anxious or depressed ❑
I am extremely anxious or depressed ❑

© 2009 EuroQol Research Foundation. EQ-5D™ is a trade mark of the EuroQol Research Foundation. UK (English) v1.2

Participant ID: ___ ___ ___ ___ ___ P

Date of visit (DD/MM/YYYY): __ __ / __ __ / __ __ __ __

- We would like to know how good or bad your health is TODAY.
- This scale is numbered from 0 to 100.
- 100 means the <u>best</u> health you can imagine.
  0 means the <u>worst</u> health you can imagine.
- Please mark an X on the scale to indicate how your health is TODAY.
- Now, write the number you marked on the scale in the box below.

YOUR HEALTH TODAY = [ ]

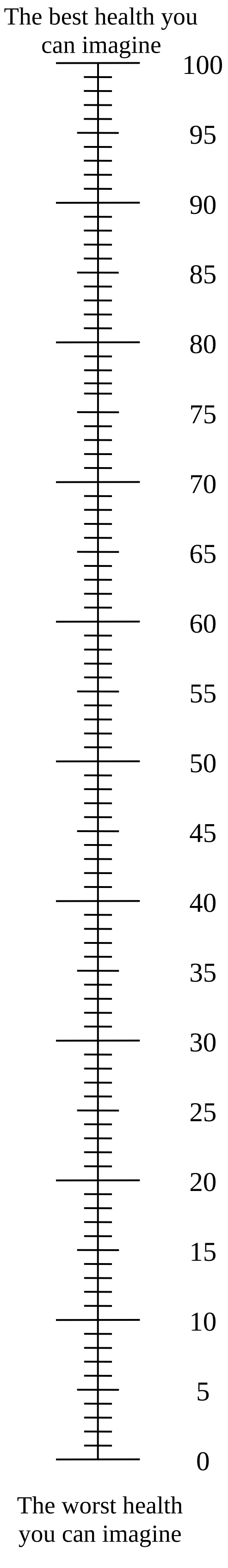

© 2009 EuroQol Research Foundation. EQ-5D™ is a trade mark of the EuroQol Research Foundation. UK (English) v1.2

Medication side-effects (GASS)

This questionnaire is about how you have been recently. It is being used to determine if you are suffering from excessive side effects from your antipsychotic medication.
Please place a tick in the column which best indicates the degree to which you have experienced the following side effects.
Also tick the **end or last** box if you found that the side effect was distressing for you.

© Waddell & Taylor, 2007

Over the past week:	*Never*	*Once*	*A few times*	*Everyday*	*Tick this box if distressing*
1. I felt sleepy during the day					
2. I felt drugged or like a zombie					
3. I felt dizzy when I stood up and/or have fainted					
4. I have felt my heart beating irregularly or unusually fast					
5. My muscles have been tense or jerky					
6. My hands or arms have been shaky					
7. My legs have felt restless and/or I couldn't sit still					
8. I have been drooling					
9. My movements or walking have been slower than usual					
10. I have had uncontrollable movements of my face or body					
11. My vision has been blurry					
12. My mouth has been dry					
13. I have had difficulty passing urine					
14 (a). I have felt like I am going to be sick or have vomited					
14 (b). I have had problems opening my bowels (constipation)					
15. I have wet the bed					
16. I have been very thirsty and/or passing urine frequently					
17. The areas around my nipples have been sore and swollen					
18. I have noticed fluid coming from my nipples					
19. I have had problems enjoying sex					
20. **Men only:** I have had problems getting an erection					

Tick yes or no for the last three months	*No*	*Yes*	*Tick this box if distressing*
21. **Women only**: I have noticed a change in my periods			
22. **Men and women**: I have been gaining weight			

Participant ID: ___ ___ ___ ___ ___ P

Date of visit (DD/MM/YYYY): __ __ / __ __ / __ __ __ __

Adherence (MARS)

Please respond to the following statements by selecting the response which best describes your behaviour or the attitude you have held toward your medication in the past week.

1. Do you ever forget to take your medication?	Yes / No
2. Are you careless at times about taking your medicine?	Yes / No
3. When you feel better, do you sometimes stop taking your medicine?	Yes / No
4. Sometimes if you feel worse when you take the medicine, do you stop taking it?	Yes / No
5. I take my medication only when I am sick.	Yes / No
6. It is unnatural for my mind and body to be controlled by medication.	Yes / No
7. My thoughts are clearer on medication.	Yes / No
8. By staying on medication I can prevent getting sick.	Yes / No
9. I feel weird, like a "zombie", on medication.	Yes / No
10.Medication makes me feel tired and sluggish.	Yes / No

© December 2000 Monash University

Participant ID: ___ ___ ___ ___ ___ P

Date of visit (DD/MM/YYYY): __ __ / __ __ / __ __ __ __

Functioning (WSAS)

People's problems sometimes affect their ability to do certain day-to-day tasks in their lives. To rate your problems look at each section and determine on the scale provided how much your problem impairs your ability to carry out the activity. This assessment is not intended to be a diagnosis. If you are concerned about your results in any way, please speak with a qualified health professional.

If you're retired or choose not have a job for reasons unrelated to your problem, tick here: ☐

0	1	2	3	4	5	6	7	8
Not at all		Slightly		Definitely		Markedly		Very severely

1	Because of my bipolar depression my ability to work is impaired. '0' means 'not at all impaired' and '8' means very severely impaired to the point I can't work.<br>***Please read the statement and enter a number from 1 to 8 in the box >***	☐
2	Because of my bipolar depression my home management (cleaning, tidying, shopping, cooking, looking after home or children, paying bills) is impaired.	☐
3	Because of my bipolar depression, my social leisure activities (with other people e.g. parties, bars, clubs, outings, visits, dating, home entertaining) are impaired.	☐
4	Because of my bipolar depression, my private leisure activities (done alone, such as reading, gardening, collecting, sewing, walking alone) are impaired.	☐
5	Because of my bipolar depression, my ability to form and maintain close relationships with others, including those I live with, is impaired.	☐

Treatment Satisfaction (TSQM, Version II)

Instructions: Please take some time to think about your level of satisfaction or dissatisfaction with the medication you are taking for your mental health. We are interested in what you think about the effectiveness, side effects, and convenience experienced when using the medication *over the last two to three weeks, or since you last used it*. For each question, please place one tick next to the response that most closely corresponds to your own experiences.

1. How satisfied or dissatisfied are you with the ability of the medication to prevent or treat the condition?

☐ Extremely Dissatisfied
☐ Very Dissatisfied
☐ Dissatisfied
☐ Somewhat Satisfied
☐ Satisfied
☐ Very Satisfied
☐ Extremely Satisfied

2. How satisfied or dissatisfied are you with the way the medication relieves symptoms?

☐ Extremely Dissatisfied
☐ Very Dissatisfied
☐ Dissatisfied
☐ Somewhat Satisfied
☐ Satisfied
☐ Very Satisfied
☐ Extremely Satisfied

3. As a result of taking this medication, do you experience any side effects at all?

☐ Yes
☐ No

4. How dissatisfied are you by side effects that interfere with your physical health and ability to function (e.g. strength, energy levels)?

☐ Extremely Dissatisfied
☐ Very Dissatisfied
☐ Somewhat Dissatisfied
☐ Slightly Dissatisfied
☐ Not at all Dissatisfied
☐ Not Applicable

"Copyright © 2006 Quintiles. All Rights Reserved."

Copyright © 2006 Quintiles. All Rights Reserved.

5. How dissatisfied are you by side effects that interfere with your mental function (e.g. ability to think clearly, stay awake)?

☐ Extremely Dissatisfied
☐ Very Dissatisfied
☐ Somewhat Dissatisfied
☐ Slightly Dissatisfied
☐ Not at all Dissatisfied
☐ Not Applicable

6. How dissatisfied are you by side effects that interfere with your mood or emotions (e.g. anxiety/fear, sadness, irritation/anger)?

☐ Extremely Dissatisfied
☐ Very Dissatisfied
☐ Somewhat Dissatisfied
☐ Slightly Dissatisfied
☐ Not at all Dissatisfied
☐ Not Applicable

7. How satisfied or dissatisfied are you with how easy the medication is to use?

☐ Extremely Dissatisfied
☐ Very Dissatisfied
☐ Dissatisfied
☐ Somewhat Satisfied
☐ Satisfied
☐ Very Satisfied
☐ Extremely Satisfied

8. How satisfied or dissatisfied are you with how easy it is to plan when you will use the medication each time?

☐ Extremely Dissatisfied
☐ Very Dissatisfied
☐ Dissatisfied
☐ Somewhat Satisfied
☐ Satisfied
☐ Very Satisfied
☐ Extremely Satisfied

"Copyright © 2006 Quintiles. All Rights Reserved."

9. How satisfied or dissatisfied are you by how often you are expected to use/take the medication?

☐ Extremely Dissatisfied
☐ Very Dissatisfied
☐ Dissatisfied
☐ Somewhat Satisfied
☐ Satisfied
☐ Very Satisfied
☐ Extremely Satisfied

10. How satisfied are you that the good things about this medication outweigh the bad things?

☐ Extremely Dissatisfied
☐ Very Dissatisfied
☐ Dissatisfied
☐ Somewhat Satisfied
☐ Satisfied
☐ Very Satisfied
☐ Extremely Satisfied

11. Taking all things into account, how satisfied or dissatisfied are you with this medication?

☐ Extremely Dissatisfied
☐ Very Dissatisfied
☐ Dissatisfied
☐ Somewhat Satisfied
☐ Satisfied
☐ Very Satisfied
☐ Extremely Satisfied

Copyright © 2006 Quintiles. All Rights Reserved.

Participant ID: ___ ___ ___ ___ ___ P

Date of visit (DD/MM/YYYY): __ __ / __ __ / __ __ __ __

Quality of Life (ICECAP-A, V2)

ABOUT YOUR OVERALL QUALITY OF LIFE

Please indicate which statements best describe your overall quality of life at the moment by placing a tick (✓) in ONE box for each of the five groups below.

1. Feeling settled and secure

I am able to feel settled and secure in **all** areas of my life	☐
I am able to feel settled and secure in **many** areas of my life	☐
I am able to feel settled and secure in **a few** areas of my life	☐
I am **unable** to feel settled and secure in **any** areas of my life	☐

2. Love, friendship and support

I can have **a lot** of love, friendship and support	☐
I can have **quite a lot** of love, friendship and support	☐
I can have **a little** love, friendship and support	☐
I **cannot** have **any** love, friendship and support	☐

3. Being independent

I am able to be **completely** independent	☐
I am able to be independent in **many** things	☐
I am able to be independent in **a few** things	☐
I am **unable** to be at all independent	☐

4. Achievement and progress

I can achieve and progress in **all** aspects of my life	☐
I can achieve and progress in **many** aspects of my life	☐
I can achieve and progress in **a few** aspects of my life	☐
I **cannot** achieve and progress in **any** aspects of my life	☐

5. Enjoyment and pleasure

I can have **a lot** of enjoyment and pleasure	☐
I can have **quite a lot** of enjoyment and pleasure	☐
I can have **a little** enjoyment and pleasure	☐
I **cannot** have **any** enjoyment and pleasure	☐

Please ensure you have only ticked ONE box for each of the five groups.

© 2010 Hareth Al-Janabi and Joanna Coast

Wellbeing (OxCAP-MH)

This questionnaire asks about your overall quality of life.

1	**Does your health in any way limit your daily activities, compared to most people of your age?**<br><br>*[Please tick one]*	☐ Always<br>☐ Most of the time<br>☐ Some of the time<br>☐ Hardly ever<br>☐ Never
2	**Are you able to meet socially with friends or relatives?**<br><br>*[Please tick one]*	☐ Always<br>☐ Most of the time<br>☐ Some of the time<br>☐ Hardly ever<br>☐ Never
3	**In the past 4 weeks, how often have you lost sleep over worry?**<br><br>*[Please tick one]*	☐ Always<br>☐ Most of the time<br>☐ Some of the time<br>☐ Hardly ever<br>☐ Never
4	**In the past 4 weeks, how often have you been able to enjoy your recreational activities?**<br><br>*[Please tick one]*	☐ Always<br>☐ Most of the time<br>☐ Some of the time<br>☐ Hardly ever<br>☐ Never
5	**How suitable or unsuitable is your accommodation for your current needs?**<br><br>*[Please tick one]*	☐ Very suitable<br>☐ Fairly suitable<br>☐ Neither suitable nor unsuitable<br>☐ Fairly unsuitable<br>☐ Very unsuitable

**6**	**Please indicate how safe you feel walking alone in the area near your home:**<br><br>*[Please tick one]*	☐ Very safe<br>☐ Fairly safe<br>☐ Neither safe nor unsafe<br>☐ Fairly unsafe<br>☐ Very unsafe
**7**	**Please indicate how likely you believe it to be that you will be assaulted in the future (including sexual and domestic assault):**<br><br>*[Please tick one]*	☐ Very likely<br>☐ Fairly likely<br>☐ Neither likely nor unlikely<br>☐ Fairly unlikely<br>☐ Very unlikely
**8**	**How likely do you think it is that you will experience discrimination?**<br><br>*[Please tick one]*	☐ Very likely (*Go to Q8a*)<br>☐ Fairly likely (*Go to Q8a*)<br>☐ Neither likely nor unlikely (*Go to Q9*)<br>☐ Fairly unlikely (*Go to Q9*)<br>☐ Very unlikely (*Go to Q9*)
**8a**	**On what grounds do you think it is likely that you will be discriminated against?**<br><br>*[Please tick up to three]*	☐ Race/ethnicity<br>☐ Gender<br>☐ Religion<br>☐ Sexual orientation<br>☐ Age<br>☐ Health or disability (incl. mental health)<br>☐ Other ………………….............…….

9	**Please indicate how strongly you agree or disagree with the following statements:**<br><br>*[Please tick one]*	Strongly Agree	Agree	Neither agree nor disagree	Disagree	Strongly disagree
**9a**	**I am free to influence decisions affecting my local area.**					
**9b**	**I am free to express my views, including political and religious views.**					
**9c**	**I am able to appreciate and value plants, animals and the world of nature.**					
**9d**	**I am able to respect, value and appreciate people around me.**					
**9e**	**I find it easy to enjoy the love, care and support of my family and/or friends.**					
**9f**	**I am free to decide for myself how to live my life.**					
**9g**	**I am able to use my imagination and to express myselfcreatively (e.g. through art, literature, music, etc.).**					
**9h**	**I have access to interesting forms of activity (or employment).**					

Health Economics Questionnaire (HEQ)

HEALTH ECONOMICS QUESTIONNAIRE (HEQ)[1] COVID-19[2]
Baseline

This questionnaire asks about your usual living situation (Section 1); employment (Section 2); income (Section 3); usual activities (Section 4); contacts with health and social services (Section 5); medication (Section 6); and COVID-19-related resource use (Section 7) over the **last 4 weeks**. If you do not know the exact answer, please give your best estimate.

1. USUAL LIVING SITUATION

Section 1 is not applicable to the ASCEND trial; please continue to Section 2

© Judit Simon/Susanne Mayer, Medical University of Vienna, 2016, 2021, 2023

2. *EMPLOYMENT*

2.1 What is your current employment status? ☐

1 Paid employment (*go to question 2.3*)
2 Self employment (*go to question 2.3*)
3 Unemployed (*go to question 2.2*)
4 Housewife/-husband (*go to question 3.1*)
5 Student (*go to question 3.1*)
6 Retired (*go to question 3.1*)
7 Voluntary employment (*go to question 3.1*)
8 Sheltered employment (*go to question 3.1*)
9 Other (please specify): ________________ (*go to question 3.1*)

2.2 ***If unemployed:***

Number of weeks unemployed over the last 4 weeks ☐ weeks
(*go to question 3.1*)

2.3 ***If in paid employment or self-employed,*** state occupation: ☐

1 Manager/administrator
2 Professional *(e.g. health, teaching, legal)*
3 Associate professional *(e.g. technical, nursing)*
4 Clerical worker /secretary
5 Skilled labourer *(e.g. building, electrical etc.)*
6 Services/sales *(e.g. retail)*
7 Factory worker
8 Other (please specify): ____________________

2.4 Do you work part-time? ☐

1 No (*go to question 2.5*)
2 Yes

If yes: How many hours do you work per week?
(*Please refer to the number of hours your contract specifies.*)

☐☐ hours

2.5 What is your personal **net** income **per month from paid work**?
(*This refers to the amount you actually receive. We are interested only in your income, i.e. exclusive of, if present, your partner's income.*)

£☐☐☐☐☐

Don't know/don't wish to reveal ☐

© Judit Simon/Susanne Mayer, Medical University of Vienna, 2016, 2021, 2023

2.6 Did health problems oblige you to be off work over the last 4 weeks? ☐

1 No (*go to question 2.7*)
2 Yes

If yes: How many days of work have you missed in the last 4 weeks?

☐☐ days

2.7 When at work, was your job performance adversely affected by health problems over the last 4 weeks? ☐

1 No (*go to question 3.1*)
2 Yes

If yes: On how many days during the last 4 weeks did you perform paid work, although you were bothered by health problems?
(*Please do not count the days on which you did not work at all because you were off sick.*)

☐☐ days

Please circle how well you performed on the days you went to work even though you were bothered by health problems.
(*1 indicates a much worse performance than usual and 10 indicates that your work was not affected.*)

1 2 3 4 5 6 7 8 9 10

much worse ... as usual

3. INCOME

3.1 Do you receive any state benefits? ☐
1 No (*go to question 3.2*)
2 Yes

If yes: What benefits are received?
(Please tick all boxes that apply.)

1 Unemployment /income support ☐
2 Sickness/disability ☐
3 Housing ☐
4 State pension ☐
5 Child benefit ☐
6 Other (please specify):____________________ ☐

3.2 What is your **main** income source? ☐
1 Salary/Wage
2 State benefits
3 Pension
4 Family support *(e.g. from spouse)*
5 Other (please specify): ____________________

3.3 What is your total personal **net** income **per month from all sources** (incl. salary/wage, state benefits, pension, family support, etc.)?
(*This refers to the amount you actually receive. We are interested only in your income, i.e. exclusive of, if present, your partner's income.*)

£☐☐☐☐☐

Don't know/don't wish to reveal ☐

4. USUAL ACTIVITIES

4.1 Did health problems influence your performance in the following activities over the last 4 weeks?

a. Personal care (*e.g. washing, dressing*) ☐

1 Performed this activity without being bothered by health problems
2 Performed this activity, although bothered by health problems
3 Did not perform this activity because of health problems
4 Did not perform this activity for reasons other than health problems

b. Household work (*e.g. cooking, cleaning*) ☐

1 Performed this activity without being bothered by health problems
2 Performed this activity, although bothered by health problems
3 Did not perform this activity because of health problems
4 Did not perform this activity for reasons other than health problems

c. Tasks outside the home (*e.g. shopping, visit to bank/post office*) ☐

1 Performed this activity without being bothered by health problems
2 Performed this activity, although bothered by health problems
3 Did not perform this activity because of health problems
4 Did not perform this activity for reasons other than health problems

d. Tasks around the home (*e.g. house maintenance, gardening, car washing*) ☐

1 Performed this activity without being bothered by health problems
2 Performed this activity, although bothered by health problems
3 Did not perform this activity because of health problems
4 Did not perform this activity for reasons other than health problems

e. Child care (*e.g. playing, taking the children to school, helping with homework*) ☐

1 Performed this activity without being bothered by health problems
2 Performed this activity, although bothered by health problems
3 Did not perform this activity because of health problems
4 Did not perform this activity for reasons other than health problems
5 Not applicable

4.2 Did other people take over or help with your usual household tasks over the last 4 weeks in connection with health problems? ☐

1 No (*go to question 5.1*)
2 Yes

If yes, what was the average number of **hours per week** you received help for over the last 4 weeks? *(More than one answer is possible.)*

Family members/friends: ☐☐☐ hours per week

Other persons receiving no pay: ☐☐☐ hours per week

Home care: ☐☐☐ hours per week

Other paid care: ☐☐☐ hours per week

5. *HEALTH AND SOCIAL CARE SERVICES*

5.1 Please list any use of **inpatient/daypatient hospital services** over the last 4 weeks
(Please enter '0' as Number of admissions if service has not been used.)

**MENTAL HEALTH CARE**		
**Service**	**Number of admissions**	**Total number of days in hospital**
Acute psychiatric ward		
Psychiatric emergency/intensive/crisis care ward		
Psychiatric long-stay ward		
Psychiatric rehabilitation ward		
**NON-MENTAL HEALTH CARE**		
**Service**	**Number of admissions**	**Total number of days in hospital**
Type of hospital department/service (e.g. oncology, surgery):____________________ Reason for admission:____________________		
Type of hospital department/service (e.g. oncology, surgery):____________________ Reason for admission:____________________		
Type of hospital department/service (e.g. oncology, surgery):____________________ Reason for admission:____________________		

5.2 Please list any use of **outpatient hospital services** (face-to-face, online, phone) over the last 4 weeks
(Please enter '0' if service has not been used.)

**Service**	**Number of times**
Psychiatric outpatient service	
Other hospital outpatient service	
A&E service	
Other (please specify):____________________	

5.3 Please list any use of **community-based day services** (face-to-face, online, phone) over the last 4 weeks
(Please enter '0' if service has not been used.)

**Service**	**Number of times**	**Average duration per service use (minutes)**
Community mental health centre		
Day care centre		
Sheltered workshop		
Specialist education		
Self-help/support group		
Other (please specify):____________________		

5.4 Please list any other use of **primary and community care services** (face-to-face, online, phone) over the last 4 weeks *(Please enter '0' if service has not been used.)*

**Service**	**Sector:** 1 = public 2 = private 3 = voluntary	**Total number of times**	**Average duration per service use (minutes)**
Psychiatrist			
Psychologist			
Primary care physician			
Primary care practice nurse			
Community/district nurse			
Community psychiatric nurse/ Case manager			
Social worker			
Occupational therapist			
Home help			
Home care worker			
Emergency services/ Ambulance			
Public health services (e.g. COVID-19 test)			
Laboratory services (e.g. COVID-19 test)			
Alternative practitioner (e.g. acupuncturist)			
Other (please specify):________________			
Other (please specify):________________			

5.5 What is the estimated distance between your usual accommodation and the primary care physician/psychiatrist you have been consulting with your mood problem over the last 4 weeks?

☐☐ miles

Total number of face-to-face visits to primary care physician/psychiatrist: ☐☐

☐ Not applicable

6. MEDICATION

7. COVID-19

Section 6 and 7 are not applicable to the ASCEnD trial, end of questionnaire.

Thank you for taking the time to complete these questionnaires.
