# Supplementary material for: Open-label randomised controlled trial of aripiprazole/sertraline combination in comparison with quetiapine for the clinical and cost-effectiveness of treatment of bipolar depression (the ASCEnD study): study protocol
Source: BMJ Open. 2026 Mar 19;16(3):e112677. doi: 10.1136/bmjopen-2025-112677 (PMC13007169; doi:10.1136/bmjopen-2025-112677)
Supplement: online supplemental appendix 10 [file bmjopen-16-3-s011.pdf]

To be printed on local headed paper

ASCEnD
Aripiprazole Sertraline Combination Effectiveness

**Participant ID** _ _ _ _ _ _

# ASCEnD-REWARD Study (Optional): Participant Instruction Guide

- A video of these instructions can be found on the ASCEnD website: https://www.ascendtrial.co.uk

- **QR code LINK to the REWARD study tasks can be found here:**

- Alternatively, a web LINK to the REWARD study tasks can be found here: https://research.sc/participant/login/161034/publicid

# 1 Overview of the REWARD study

## 1.1 What is the purpose of the REWARD study?

- The ASCEnD trial has the goal of comparing the effectiveness of an aripiprazole/sertraline combination with quetiapine. The REWARD study aims to take our knowledge of the treatments' effectiveness a step further by investigating why this may differ.
- By taking part in the REWARD study, you will be able to improve the understanding of psychological processes by which the treatments work, which are currently not fully understood.
- The psychological process that the REWARD study will assess is reward processing. Reward processing, including the ability to feel pleasure and to learn from positive outcomes, is known to be impacted in depression. Improved reward processing is a potential mechanism for the treatments proposed in ASCEnD, due to their effect on the brain chemicals that allow reward processing.
- Understanding the mechanism of the treatments is a crucial step to improve the way bipolar depression is treated. It helps identification of which treatment may benefit a patient early during the treatment, and even before the treatment is started. This allows a more targeted, individualised approach to treatment and helps reduce the time spent by patients using a less effective treatment.

## 1.2 What does the REWARD study involve?

- The REWARD study will involve three sessions. Each session should not take longer than 30 minutes.
- For each session, you will complete the same decision-making task and questionnaire online, on the data collection platform GORILLA (gorilla.sc)
- You can access and complete the sessions on a PC, tablet, or smartphone with an internet connection**. Please complete all sessions from the same type of device where possible**.

- To access the study, you will need:
    - Web link or QR code (see top of this instruction).
    - Your unique ASCEnD participant ID (see top of this instruction).
    - Your month and year of birth.

*Please note that if you are randomised to the same medication you are currently taking (aripiprazole or quetiapine), you will not be able to take part in the study. Sertraline is irrelevant for taking part in the study.*

## 1.3 Timing of sessions

- **SESSION 1** – You will complete SESSION 1 *before* starting your prescribed medication (quetiapine or aripiprazole). **Please do not delay starting your prescribed medication only because you are not able to complete the task.**

*Please note that if you are unable to complete SESSION 1 before starting your prescribed medication, you will not be able to complete SESSION 2 and 3.*

You will be told during the weekly ASCEnD phone calls when it is time to complete a new session (SESSION 2 and 3). **Please do not complete a new session until this is mentioned during the phone calls.**

- **SESSION 2** – You will complete SESSION 2 between 2 and 4 weeks after you started your prescribed medication.

- **SESSION 3** – You will complete SESSION 3 between 12 and 14 weeks after you started your prescribed medication.

# 2 Instructions

## 2.1 Accessing a session on the GORILLA data-collection platform

- You can access the task and questionnaire by **scanning the QR code** or **typing the web link** onto your browser bar. **You can find the QR code and link at the top of these instructions**.
- You will then be prompted to provide an ID. Please **type your unique ASCEnD Participant ID** that is noted at the top of these instructions into the response bar shown below:

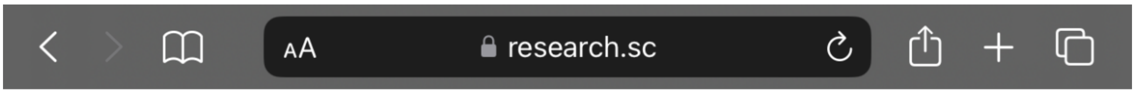


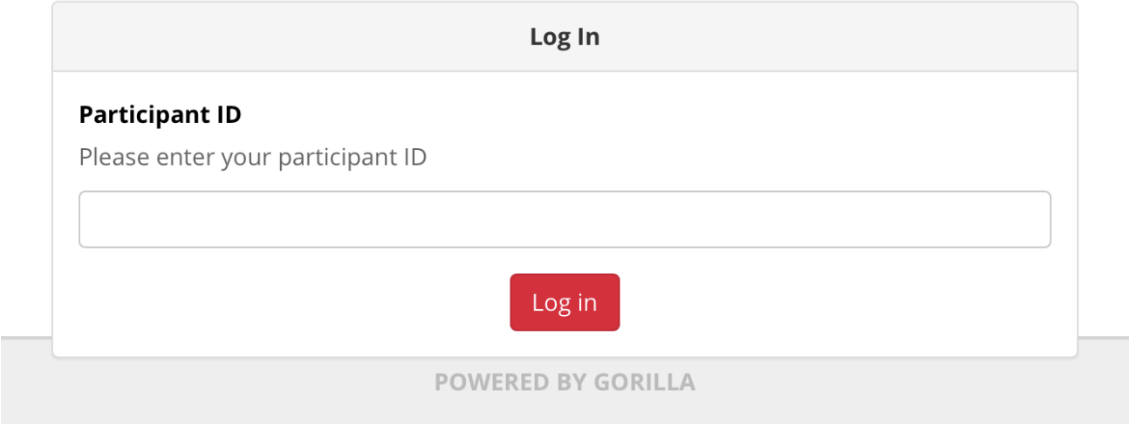


- **The same link/QR code and your ASCEnD participant ID will allow you to access all three REWARD task sessions.**
- If at any point you can't find your link/QR code or ASCEnD participant ID, please ask for this to be shared with you again during your weekly phone calls.
- If you get an error message, please double-check the spelling of your ASCEnD participant ID (ensuring no blank spaces) and try again. If this does not work, please request help during your next weekly phone call.

## 2.2 Decision-Making task

*Below is an outline of the decision-making task. Please note that there will be instructions to the task on the GORILLA website for you to read before completing each session. The information below is only provided as an overview of what to expect from the task.*

- You will see two different shapes appearing on each side of a cross in the centre of the screen multiple times, and you will be asked to choose one of them each time. For example:

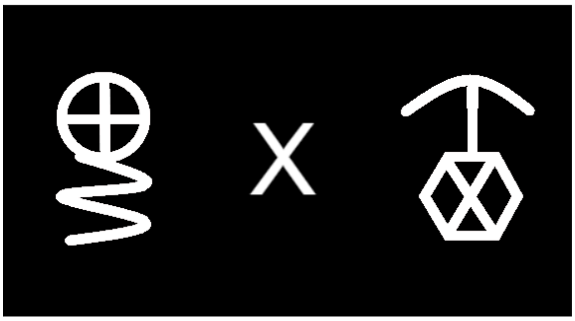

- **Please note that the shapes may change sides each time they appear. The side doesn't matter, what matters is the shape itself.**
- Some of the shapes you will see will be associated with a higher chance of winning points and some with a higher chance of losing points. Your job is to collect as many points as possible (win more and lose less).

To choose a shape you can:

- On a touch-screen device: Tap on the screen directly on the shape of your choice.
- On a computer: Use the mouse to point and click on the shape of your choice using the **left** button on your mouse.
- **Although you may be able to predict which shape is most likely to win points or lose points, it won't *always* be the case that a particular shape leads to a win or a loss**.

- **You need to judge which shape is *most likely* to result in a win or a loss (for example, 7 out of 10 times).** Below are examples of the outcomes you may get after each choice depending on which shape you choose:

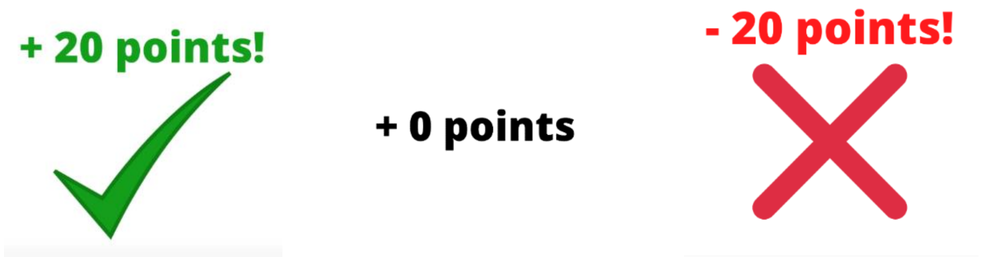


- There will be 2 blocks (10 minutes each) to complete. If you need a break, you can do so in between blocks (up to 5 minutes). **Please note that it is not possible to stop the task during each block, so please only take a break between blocks.**
- **If you are completing the task on your smartphone, please switch off notifications and incoming calls during this task where possible.**

## 2.3 Questionnaire

Once you get to the end of the decision-making task, you will be asked to click the "NEXT" button to access the questionnaire. The questionnaire consists of 24 statements, and you will be asked to rate how much each statement is true for you on a scale from *"Very true for me"* to *"Very false for me".*

## 2.4 Ending a session

When you reach the **end of a session**, you will see a screen like the one over the page. Please close the webpage**. Your progress will be saved automatically**.

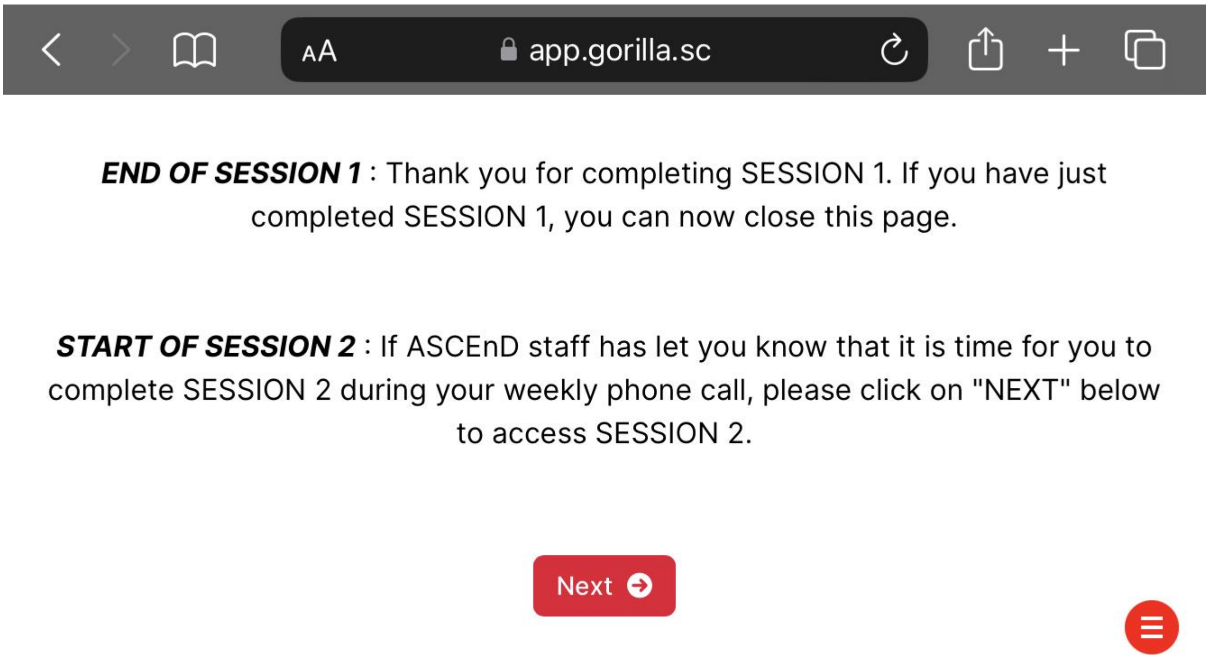


## 2.5 Starting a new session (SESSION 2 and 3)

During your weekly phone call, you will be told when it is time to complete a new session (SESSION 2 and 3**).** You can access these using the same link/QR code and ASCEnD participant ID you used for previous sessions. You will start from the same screen you saw at the end of your previous session. **Please click on the "NEXT" button** to move on to the session you want to complete.

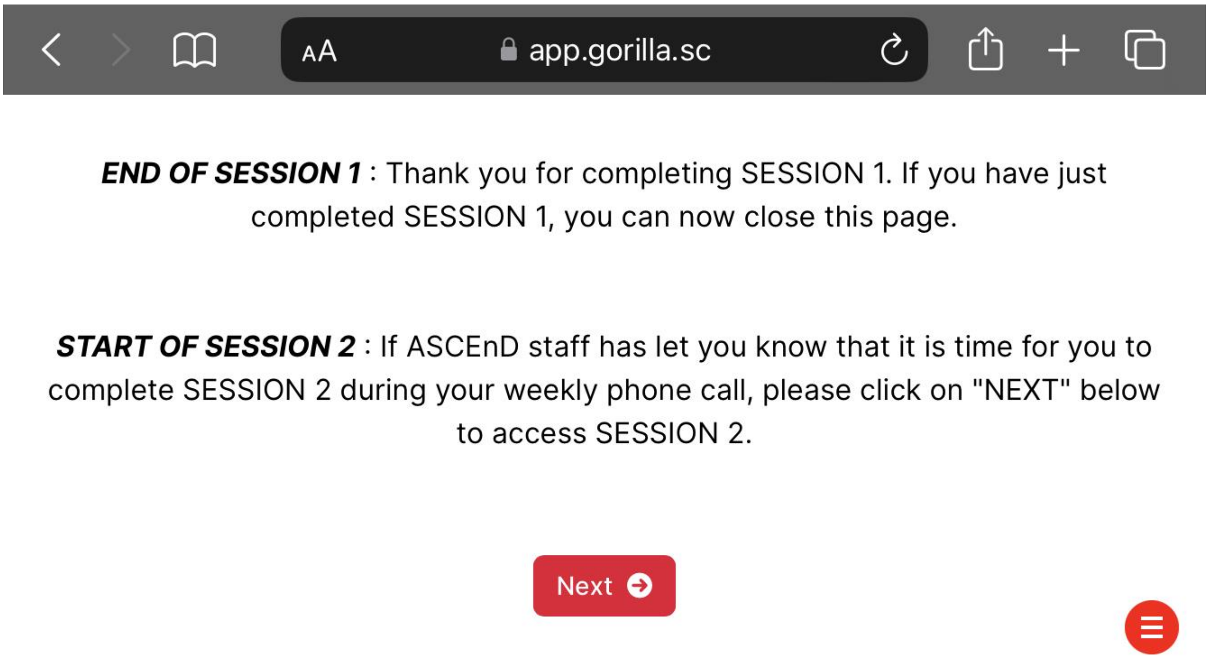

## 2.6 End of study (end of SESSION 3)

When you reach the end of SESSION 3, you will have completed the REWARD study. You will see the screen shown below:

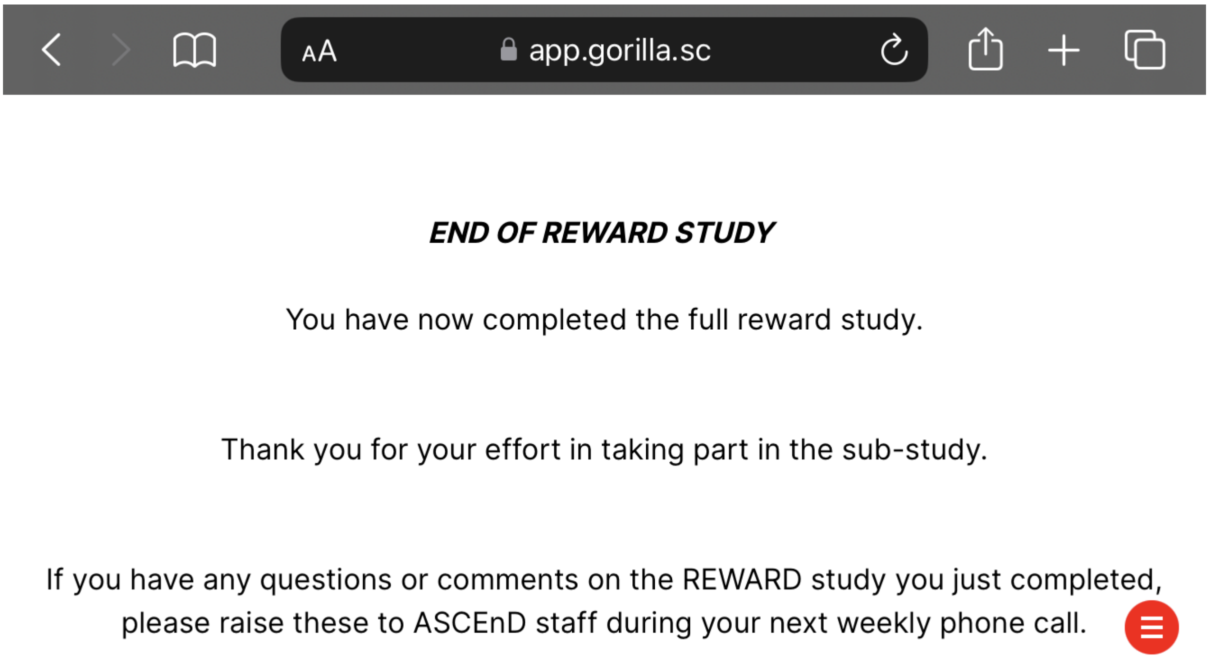


*Thank you for taking the time to read the instructions to the REWARD study and for your interest in taking part. If you have any questions or comments related to the REWARD study, please ask during your next weekly call.*
