# Supplementary material for: Open-label randomised controlled trial of aripiprazole/sertraline combination in comparison with quetiapine for the clinical and cost-effectiveness of treatment of bipolar depression (the ASCEnD study): study protocol
Source: BMJ Open. 2026 Mar 19;16(3):e112677. doi: 10.1136/bmjopen-2025-112677 (PMC13007169; doi:10.1136/bmjopen-2025-112677)
Supplement: online supplemental appendix 11 [file bmjopen-16-3-s012.pdf]

ASCEnD

Aripiprazole Sertraline Combination Effectiveness

Participant Diary

Information and Instructions

- This diary is for you to complete and keep. It will help you remember information that we need to collect from you during the study.
- Please have this diary at hand for Research Assistant (RA) phone calls.
- Please remember to log into the online system Red Pill to complete the study questionnaires when prompted to.

You started the ASCEND study on: _ _ / _ _ / 20_ _

[Site staff to input date of randomisation in space above]

Contents

My Study Information 3
Dose Log.. 4
Side Effects 28
Any Other Prescribed Medication 30

My Study Information

I have been prescribed: ☐ Sertraline/Aripiprazole

☐ Quetiapine

You may find it helpful to use this section to note down the names and contact information of people involved in this study.

My study doctor(s) is:_______________________________________

The Research Assistants are:________________________________

Other members of my study team (e.g. Research Nurse) are:

____

____

____

Study website: www.research.ncl.ac.uk/ascend

Dose Log

Please use the table below to record the study medication you have taken from the day after you are sorted into your study group. Please also record if you missed a dose for any reason.

Week	Day	Date	Study Medication Taken?	Dose taken (mg)	Missed Dose?	Reason for Missed Dose/Comments
**Example**		01/01/2023	**Yes**	10mg	No	
1	1					
	2					
	3					
	4					
	5					
	6					
	7					
**Did you have any side effects? Any comments?**						

Week	Day	Date	Study Medication taken?	Dose taken (mg)	Missed Dose?	Reason for Missed Dose/Comments
2	8					
	9					
	10					
	11					
	12					
	13					
	14					

Did you have any side effects? Any comments?

Week	Day	Date	Study Medication taken?	Dose taken (mg)	Missed Dose?	Reason for Missed Dose/Comments
3	15					
	16					
	17					
	18					
	19					
	20					
	21					

**Did you have any side effects? Any comments?**

Week	Day	Date	Study Medication taken?	Dose taken (mg)	Missed Dose?	Reason for Missed Dose/Comments
4	**22**					
	23					
	24					
	25					
	26					
	27					
	28					

**Did you have any side effects? Any comments?**

Week	Day	Date	Study Medication taken?	Dose taken (mg)	Missed Dose?	Reason for Missed Dose/Comments
5	**29**					
	30					
	31					
	32					
	33					
	34					
	35					
**Did you have any side effects? Any comments?**						

Week	Day	Date	Study Medication taken?	Dose taken (mg)	Missed Dose?	Reason for Missed Dose/Comments
6	**36**					
	37					
	38					
	39					
	40					
	41					
	42					

**Did you have any side effects? Any comments?**

Week	Day	Date	Study Medication taken?	Dose taken (mg)	Missed Dose?	Reason for Missed Dose/Comments
7	**43**					
	44					
	45					
	46					
	47					
	48					
	49					

**Did you have any side effects? Any comments?**

Week	Day	Date	Study Medication taken?	Dose taken (mg)	Missed Dose?	Reason for Missed Dose/Comments
8	**50**					
	51					
	52					
	53					
	54					
	55					
	56					
**Did you have any side effects? Any comments?**						

Week	Day	Date	Study Medication taken?	Dose taken (mg)	Missed Dose?	Reason for Missed Dose/Comments
9	**57**					
	58					
	59					
	60					
	61					
	62					
	63					

**Did you have any side effects? Any comments?**

Week	Day	Date	Study Medication taken?	Dose taken (mg)	Missed Dose?	Reason for Missed Dose/Comments
10	**64**					
	65					
	66					
	67					
	68					
	69					
	70					

**Did you have any side effects? Any comments?**

Week	Day	Date	Study Medication taken?	Dose taken (mg)	Missed Dose?	Reason for Missed Dose/Comments
11	**71**					
	72					
	73					
	74					
	75					
	76					
	77					

**Did you have any side effects? Any comments?**

Week	Day	Date	Study Medication taken?	Dose taken (mg)	Missed Dose?	Reason for Missed Dose/Comments
12	**78**					
	79					
	80					
	81					
	82					
	83					
	84					

**Did you have any side effects? Any comments?**

Week	Day	Date	Study Medication taken?	Dose taken (mg)	Missed Dose?	Reason for Missed Dose/Comments
13	**85**					
	86					
	87					
	88					
	89					
	90					
	91					
**Did you have any side effects? Any comments?**						

Week	Day	Date	Study Medication taken?	Dose taken (mg)	Missed Dose?	Reason for Missed Dose/Comments
14	**92**					
	93					
	94					
	95					
	96					
	97					
	98					

**Did you have any side effects? Any comments?**

Week	Day	Date	Study Medication taken?	Dose taken (mg)	Missed Dose?	Reason for Missed Dose/Comments
15	**99**					
	100					
	101					
	102					
	103					
	104					
	105					

**Did you have any side effects? Any comments?**

Week	Day	Date	Study Medication taken?	Dose taken (mg)	Missed Dose?	Reason for Missed Dose/Comments
16	**106**					
	107					
	108					
	109					
	110					
	111					
	112					
**Did you have any side effects? Any comments?**						

Week	Day	Date	Study Medication taken?	Dose taken (mg)	Missed Dose?	Reason for Missed Dose/Comments
17	**113**					
	114					
	115					
	116					
	117					
	118					
	119					

**Did you have any side effects? Any comments?**

Week	Day	Date	Study Medication taken?	Dose taken (mg)	Missed Dose?	Reason for Missed Dose/Comments
18	**120**					
	121					
	122					
	123					
	124					
	124					
	125					

**Did you have any side effects? Any comments?**

Week	Day	Date	Study Medication taken?	Dose taken (mg)	Missed Dose?	Reason for Missed Dose/Comments
19	**126**					
	127					
	128					
	129					
	130					
	131					
	132					

**Did you have any side effects? Any comments?**

Week	Day	Date	Study Medication taken?	Dose taken (mg)	Missed Dose?	Reason for Missed Dose/Comments
20	**133**					
	134					
	135					
	136					
	137					
	138					
	139					

**Did you have any side effects? Any comments?**

Week	Day	Date	Study Medication taken?	Dose taken (mg)	Missed Dose?	Reason for Missed Dose/Comments
21	**140**					
	141					
	142					
	143					
	144					
	145					
	146					
**Did you have any side effects? Any comments?**						

Week	Day	Date	Study Medication taken?	Dose taken (mg)	Missed Dose?	Reason for Missed Dose/Comments
22	**147**					
	148					
	149					
	150					
	151					
	152					
	153					
**Did you have any side effects? Any comments?**						

Week	Day	Date	Study Medication taken?	Dose taken (mg)	Missed Dose?	Reason for Missed Dose/Comments
23	**154**					
	155					
	156					
	157					
	158					
	159					
	160					

**Did you have any side effects? Any comments?**

Week	Day	Date	Study Medication taken?	Dose taken (mg)	Missed Dose?	Reason for Missed Dose/Comments
24	**161**					
	162					
	163					
	164					
	165					
	166					
	167					

**Did you have any side effects? Any comments?**

Side Effects

If you experience any symptoms whilst taking the study medication, please enter the details in the table below. The research assistants will ask you about these during their calls.

Symptom	Description of symptoms	Start Date	End Date
e.g. Headache	e.g. pressure pain at the back of the head	01/02/2023	02/02/2023

Symptom	Description of symptoms	Start Date	End Date

Any Other Prescribed Medication

Please enter details in the table below if you take any other prescribed medication. Do not record any over the counter medications, herbal medications or recreational drugs you have taken. The research assistants will ask you about your prescribed medications during their calls.

Drug Name	Dose Taken	How often do you take it?	Why do you take it?	What date did you start taking it?	What date did you stop taking it?

Drug Name	Dose Taken	How often do you take it?	Why do you take it?	What date did you start taking it?	What date did you stop taking it?
