# Supplementary material for: Open-label randomised controlled trial of aripiprazole/sertraline combination in comparison with quetiapine for the clinical and cost-effectiveness of treatment of bipolar depression (the ASCEnD study): study protocol
Source: BMJ Open. 2026 Mar 19;16(3):e112677. doi: 10.1136/bmjopen-2025-112677 (PMC13007169; doi:10.1136/bmjopen-2025-112677)
Supplement: online supplemental appendix 12 [file bmjopen-16-3-s013.pdf]

To be printed on local headed paper

Aripiprazole/Sertraline combination: clinical and cost-effectiveness in comparison with Quetiapine for the treatment of bipolar depression. An open label randomised controlled trial.

Participant Consent Form

Site Name: [local site to add details] Site Number: [local site to add details]

Name of Principal Investigator: [local site to add details]

Participant Identification Number: _________________

Information for the participant

Please read each statement carefully and then write your initials in the box next to each statement if you agree. If you do not understand a statement, please ask the person helping you complete this form to explain.

SECTION A: TAKING PART		Please initial here if you agree:
1.	I have read and understood the ASCEnD Patient Information Sheet version____ dated_________________. I have had the opportunity to consider the information, ask questions and have had these answered satisfactorily.	☐
2.	I understand that my participation in this study is voluntary and that I am free to withdraw at any time without needing to provide a reason and that doing so will not affect my medical care or legal rights. I understand that if I withdraw from the study, the information collected from me until that point will be retained and used.	☐
3.	I understand that where relevant to my taking part in this study, sections of my medical records and data collected about me will be accessed or viewed by authorised individuals from my local NHS Trust, Cumbria, Northumberland, Tyne and Wear NHS Foundation Trust, Newcastle Clinical Trials Unit, Newcastle University, the collaborating institutions described in the Patient Information Sheet and if required, the regulatory authorities. I give these individuals permission to view my information.	☐

Please continue on the next page

4.	I understand that the information I provide, and which is collected about me during the study, will be kept confidential and stored securely for 5 years after the end of the study, at which time it will be safely destroyed.	☐
5.	I understand that the information collected about me may be used to support other research in the future and may be shared anonymously with other researchers both within and outside of the UK. I understand that I will not be directly identified in any published results.	☐
6.	I understand that I will be contacted via email and text message to complete questionnaires during this study and via telephone or video call to speak with study research assistants.	☐
7.	I understand that my GP and care team will be informed that I am taking part in this study.	☐
8.	I agree to take part in the ASCEnD study.	☐

SECTION B: For female participants of childbearing potential only

Please initial here if you agree:

1.	I understand that the drugs used in this study should only be used in pregnancy if the benefits are greater than the potential risks. I have had the chance to discuss the risk that I may become pregnant whilst taking these drugs and of discussing the potential risk the drugs may have if I do become pregnant. I am aware that a pregnancy test may be required if I am a woman, not post-menopausal and not using highly effective contraception.	☐

SECTION C: OPTIONAL

Please initial here if you agree or leave blank if you do not agree:

1.	I agree to be contacted by email or telephone to participate in an interview (this will take place over the phone, video call or if requested, face-to-face). The interview will be recorded by the qualitative research team at Keele University. I understand that my information will be stored securely and confidentiality at Keele University for this purpose and that what I say in my interview may be published, but I will not be identified personally. I understand that the transcription may be done by researchers based at Keele University in the UK or by a transcription company based in the USA.	☐

2.	I agree to take part in the optional REWARD study depending on my randomisation outcome. I understand that I will complete task questionnaires via the online GORILLA data collection platform and this data will be stored securely and confidentially here and on Newcastle University's One Drive. I understand I will not be personally identified following any publication.	☐
3.	I agree for a copy of my consent form to be sent to Newcastle Clinical Trials Unit for review.	☐
4.	I agree for contact between my informal carer (e.g. my spouse, family member or friend) and the study team and for my informal carer to complete study questionnaires for the ASCEnD study if they wish.	☐

Name of participant *(please print)*	Date *(DD/MMM/YYYY)*	Signature
Name of person seeking consent *(please print)*	Date *(DD/MMM/YYYY)*	Signature

Information for the person seeking consent

If the participant consents, please ensure that they have initialled each box in section A and B (if appropriate) and have signed where indicated previously. Please also ensure that you have signed in the space underneath the participant.

Please file the original consent form in your investigator site file and make two copies – one for the participant's medical records, and one to give to the participant.

If the participant has initialled point 3 in section C, please also send a copy of the completed consent form using secure email to nctu.ascend.conf@nhs.net. If they have not initialled this box, instead please complete a consent proforma and send this to nctu.ascend.conf@nhs.net, ensuring none of the participant's personal details are written on the form.
