# Supplementary material for: Open-label randomised controlled trial of aripiprazole/sertraline combination in comparison with quetiapine for the clinical and cost-effectiveness of treatment of bipolar depression (the ASCEnD study): study protocol
Source: BMJ Open. 2026 Mar 19;16(3):e112677. doi: 10.1136/bmjopen-2025-112677 (PMC13007169; doi:10.1136/bmjopen-2025-112677)
Supplement: online supplemental appendix 13 [file bmjopen-16-3-s014.pdf]

To be printed on local headed paper

Aripiprazole Sertraline Combination Effectiveness

**A**ripiprazole/**s**ertraline **c**ombination: clinical and cost-**e**ffectiveness in comparison with quetiapine for the treatment of bipolar **d**epression. An open label randomised controlled trial.

# Informal Carer Consent Form

Site Name: [local site to add details] Site Number: [local site to add details]

Name of Principal Investigator: [local site to add details]

Participant Identification Number: ________________

## Information for the participant

Please read each statement carefully and then write your initials in the box next to each statement if you agree. If you do not understand a statement, please ask the person helping you complete this form to explain.

| | | Please initial here if you agree: |
|---|---|---|
| 1. | I have read and understood the ASCEnD Informal Carer Information Sheet version____________ dated_________________. I have had the opportunity to consider the information, ask questions and have had these answered satisfactorily. | ☐ |
| 2. | I understand that my participation in this study is voluntary and that I am free to withdraw at any time without needing to provide a reason and that doing so will not affect my legal rights. I understand that if I withdraw from the study, the information collected from me until that point will be retained and used. | ☐ |
| 3. | I understand that where relevant to my taking part in this study, any personal details collected about me may be accessed or viewed by authorised individuals from my local NHS Trust, Cumbria, Northumberland, Tyne and Wear NHS Foundation Trust, Newcastle Clinical Trials Unit, Newcastle University, the collaborating institutions described in the Information Sheet and if required, the regulatory authorities. I give these individuals permission to view my information. | ☐ |

**Please continue on the next page**

4. I understand that the information I provide, and which is collected about me during the study, will be kept confidential and stored securely for 5 years after the end of the study, at which time it will be safely destroyed. ☐

5. I understand that the information collected about me may be used to support other research in the future and may be shared anonymously with other researchers both within and outside of the UK. I understand that I will not be directly identified in any published results. ☐

6. I understand that I will be contacted via email and text message to complete questionnaires during this study. ☐

7. I agree to take part in the ASCEnD study. ☐

| ________________ | ________________ | ________________ |
| --- | --- | --- |
| Name of participant<br>*(please print)* | Date<br>*(DD/MMM/YYYY)* | Signature |
| ________________ | ________________ | ________________ |
| Name of person seeking consent<br>*(please print)* | Date<br>*(DD/MMM/YYYY)* | Signature |

## Information for the person seeking consent

**If the participant consents, please ensure that they have initialled each box and signed where indicated above. Please also ensure that you have signed in the space underneath the participant.**

Please file the original consent form in your investigator site file and give a copy to the participant.
