# Supplementary material for: Open-label randomised controlled trial of aripiprazole/sertraline combination in comparison with quetiapine for the clinical and cost-effectiveness of treatment of bipolar depression (the ASCEnD study): study protocol
Source: BMJ Open. 2026 Mar 19;16(3):e112677. doi: 10.1136/bmjopen-2025-112677 (PMC13007169; doi:10.1136/bmjopen-2025-112677)
Supplement: Supplementary file 15 [file bmjopen-2025-112677.reviewer_comments.pdf]

# PEER REVIEW HISTORY

BMJ Open publishes all reviews undertaken for accepted manuscripts. Reviewers are asked to complete a checklist review form and are provided with free text boxes to elaborate on their assessment. These free text comments are reproduced below.

## ARTICLE DETAILS

### Title (Provisional)

Study protocol for an open-label randomised controlled trial of aripiprazole/sertraline combination in comparison with quetiapine for the clinical and cost-effectiveness of treatment of bipolar depression (the ASCEnD study).

### Authors

Azim, Lumbini; Al-Ashmori, Sarah; Butcher, Chrissie; Cipriani, Andrea; Chew-Graham, Carolyn; Clare, Emily; Clark, Emma; Cole, Michael; Carella, Susanna; Dixon, Lyndsey; Evans, Jonathan; Gergel, Tania; Gibson, John; Hancock, Helen C; Hoppe, Isobel; Kessler, David; Kabir, Thomas; Lewis, Glyn; Mathias, Ayesha; Morris, Richard; nixon, neil; Simon, Judith; Teare, Dawn; Lived Experience Advisory Panel, The; Wahba, Mourad; Wall, Lauren; Walmsley, Zoe; Wienand, Dennis; Wolstenhulme, Faye; Ahuja, Niraj; Watson, Stuart

---

## VERSION 1 - REVIEW

---

| | |
|---|---|
| **Reviewer** | **1** |
| **Name** | **Hashimoto, Kenji** |
| **Affiliation** | **Chiba University Center for Forensic Mental Health** |
| **Date** | **11-Dec-2025** |
| **COI** | **None** |

---

In this study, the authors aim to evaluate the clinical and cost-effectiveness of aripiprazole–sertraline compared with quetiapine, offering potential advantages in treatment acceptability, accessibility, tolerability, and clinical outcomes for bipolar depression. The trial is well designed, and the manuscript is clearly written. I have no major concerns regarding the study; however, I would like to raise one minor point related to the choice of aripiprazole–sertraline.

Minor comment:

This trial uses a combination of aripiprazole and sertraline. However, the rationale for selecting sertraline among the various available SSRIs is not clearly explained. Endoplasmic reticulum (ER) stress and the resulting mitochondrial dysfunction are increasingly recognized as contributing to the pathophysiology of bipolar depression. Considering the role of sigma-1 receptors in mitigating ER stress, SSRIs with sigma-1 receptor agonist properties (e.g., fluvoxamine, fluoxetine, escitalopram) might theoretically offer additional mechanistic

advantages. Clarifying why sertraline was chosen over other SSRIs with sigma-1 receptor activity would strengthen the justification for the intervention.

---

## VERSION 1 - AUTHOR RESPONSE

1. **Comment Reviewer 1** This trial uses a combination of aripiprazole and sertraline. However, the rationale for selecting sertraline among the various available SSRIs is not clearly explained. Endoplasmic reticulum (ER) stress and the resulting mitochondrial dysfunction are increasingly recognized as contributing to the pathophysiology of bipolar depression. Considering the role of sigma-1 receptors in mitigating ER stress, SSRIs with sigma-1 receptor agonist properties (e.g., fluvoxamine, fluoxetine, escitalopram) might theoretically offer additional mechanistic advantages. Clarifying why sertraline was chosen over other SSRIs with sigma-1 receptor activity would strengthen the justification for the intervention.
    a. **Response** Working in neuroscience in Newcastle, England makes it impossible not to closely follow the mitochondrial story. Nonetheless we did not feel in 2021 when the proposal was submitted that there was sufficient data to be overly considerate of sigma-1 receptor activity in the selection of antidepressant. As such, sertraline was chosen to combine with aripiprazole due to its strong safety profile, a favourable pharmacokinetic profile, limited interactions with most psychotropics and lack of adverse interaction with aripiprazole and risk of cardiac arrythmias.

We hope that these revisions adequately address the editorial and reviewer comments. We would be grateful for your further consideration of the revised manuscript.
